# The Fermi–Dirac distribution provides a calibrated probabilistic output for binary classifiers

**DOI:** 10.1073/pnas.2100761118

**Published:** 2021-08-19

**Authors:** Sung-Cheol Kim, Adith S. Arun, Mehmet Eren Ahsen, Robert Vogel, Gustavo Stolovitzky

**Affiliations:** ^a^IBM Research, IBM Thomas J. Watson Research Center, Yorktown Heights, NY 10598;; ^b^Gies College of Business, University of Illinois at Urbana-Champaign, Urbana, IL 61820

**Keywords:** binary classification, ensemble learning, machine learning, calibrated probability, Fermi–Dirac distribution

## Abstract

Binary classification is one of the central problems in machine-learning research and, as such, investigations of its general statistical properties are of interest. We studied the ranking statistics of items in binary classification problems and observed that there is a formal and surprising relationship between the probability of a sample belonging to one of the two classes and the Fermi–Dirac distribution determining the probability that a fermion occupies a given single-particle quantum state in a physical system of noninteracting fermions. Using this equivalence, it is possible to compute a calibrated probabilistic output for binary classifiers. We show that the area under the receiver operating characteristics curve (AUC) in a classification problem is related to the temperature of an equivalent physical system. In a similar manner, the optimal decision threshold between the two classes is associated with the chemical potential of an equivalent physical system. Using our framework, we also derive a closed-form expression to calculate the variance for the AUC of a classifier. Finally, we introduce FiDEL (Fermi–Dirac-based ensemble learning), an ensemble learning algorithm that uses the calibrated nature of the classifier’s output probability to combine possibly very different classifiers.

## Significance

While it would be desirable that the output of binary classification algorithms be the probability that the classification is correct, most algorithms do not provide a method to calculate such a probability. We propose a probabilistic output for binary classifiers based on an unexpected mapping of the probability of correct classification to the probability of occupation of a fermion in a quantum system, known as the Fermi–Dirac distribution. This mapping allows us to compute the optimal threshold to separate predicted classes and to calculate statistical parameters necessary to estimate confidence intervals of performance metrics. Using this mapping we propose an ensemble learning algorithm. In short, the Fermi–Dirac distribution provides a calibrated probabilistic output for binary classification.

Binary classification is the task of predicting the binary categorical label of each item in a set of items that belong to one of two categories (1). Typically, this prediction is made using a function, known as a classifier, which learns from examples taken from a training dataset containing items of both classes of interest. This classifier is subsequently used to predict the labels of previously unseen items contained in a new dataset.

Binary classification has a remarkably broad range of applications in fields such as biomedicine (2), economics (3), finance (4), astronomy (5), advertisement (6), and manufacturing (7). Problems addressed in these areas with greater or lesser success include predicting antibacterial activity of molecules (8), diagnosing breast cancer from mammography studies (9), detecting skin cancer from dermoscopy images (10), predicting Alzheimer’s disease onset from linguistic markers (11), classifying hand gestures from wearable device signals (12), identifying lunar craters from images (13), deciding whether a judge should have defendants wait for trial under bail at home or in jail (14), choosing whether to approve a loan to a client (15), predicting corporate financial distress (16), and determining whether a given semiconductor manufacturing process will lead to a faulty product (17).

This great diversity of applications has spurred a considerable amount of work devoted to the development of classification methods. Despite substantial theoretical progress that led to increased predictive power, there is concern that methods optimized under narrow theoretical contexts may not lead to performance generalization (18) and that the emphasis of research on prediction models should perhaps shift to other issues such as model interpretation and independent validation (19). Accordingly, in this paper we address four generic problems arising in any classification task: 1) We develop a calibrated probabilistic interpretation of the output of a classification pipeline, independent of the classification method used; 2) we show how to use this probabilistic interpretation to optimally choose a threshold that separates predicted classes; 3) we introduce an analytical way to compute the confidence interval of the most popular classification performance metric (the area under the receiver operating characteristics curve [AUC]), which uses only the available information rather than ad hoc hypotheses about the classifier; and 4) we address the issue of performance generalization by developing an ensemble approach that, rather than relying on the generalization ability of any individual method, leverages the ability of many methods to compensate each other’s deficiencies and get a performance that is often better than the best in the ensemble. To achieve these objectives we advance an unexpected equivalence between the probabilistic description of fermions in quantum statistical mechanics and the probability of correct classification of items in typical classification problems. The validity of the probabilistic aspects of fermionic systems under general conditions renders the equivalent results in the world of classification to be quite robust and independent of any individual classification method.

In a binary classification problem, the two classes to be predicted are usually denoted as the negative and the positive class. While this distinction is arbitrary, the positive class is usually chosen as the class that is more costly to misclassify. For example, in cancer screening, failing to detect patients with cancer is more costly than failing to detect disease-free subjects, and therefore cancer patients are usually assigned to be in the positive class. We use this convention in this paper. A binary classifier typically assigns a score $s_{i}$ to a given item i that can be a proxy to the confidence assigned by the classifier that the item belongs to the positive class y=1 or to the cost of misclassification of that sample. In general, the probability density of these scores will depend on the class y and is known as the class-conditioned score density P(s|y), which is not known a priori and is different for each classifier. Some authors have proposed to fit Gaussian distributions to the scores resulting from specific classifiers (20), but better results have resulted from allowing more flexibility in fitting class-conditioned densities (21). The score outputted by different classifiers can be binarized using a decision threshold in such a way that we can assign samples with scores above/below that threshold to the positive/negative class. The optimal decision threshold will depend on the class-conditioned score density and is usually chosen empirically or learned during training.

The class-conditioned density can be used to compute the posterior probability P(y|s) of the class y given the score s assigned to an item. Having a well-calibrated probability that a given item belongs to the positive class can be very useful, for example, when the result of a classifier must be merged with other classifiers in an ensemble (22, 23) or when the probability of the class assignment of a classifier needs to be combined with other probabilistic elements into a complex decision. However, most classifiers produce a score with unknown probability density from which a posterior class probability cannot be recovered. Some authors have proposed to train the posterior probability simultaneously with the classifier (24), and others have proposed to train the parameters of a logistic function that depends on the score of a previously trained classifier (25). In these methods, as the probability is trained in the training set, there is some risk of overfitting to the probabilistic output. The alternative of keeping a holdout set and using cross-validation is relatively successful (25). These strategies are feasible only if there is a sufficient amount of labeled data for the problem at hand. In cases where the amount of labeled data is not enough to train the classifier and the posterior probability, other methods are desirable.

One of the most popular metrics to measure the performance of a binary classifier is the AUC (26). To calculate the AUC of a classifier, it is necessary to rank the items using the scores assigned to each item by the classifier. Thus, the AUC of a classifier is invariant with respect to any monotonic transformation of scores. It follows that what is important in calculating the AUC is the relative ranking of an item to other items rather than the actual score assigned to an item. When the number of items in the test set increases, the AUC of a classifier asymptotically approaches the probability that the classifier assigns a randomly chosen positive sample a higher score than a randomly chosen negative sample (27). The AUC is a threshold-independent way of calculating the performance of a classifier. To predict whether items are positive or negative we need to choose a decision threshold and assign items with scores above this threshold to the positive class and items below it to the negative class. In these cases the balanced accuracy, defined as the average of the sensitivity and specificity, is a popular metric for model evaluation.

Assuming that a classifier is better than random, ranking N classified items in decreasing order from higher to lower scores will lead to positive samples having predominantly low ranks (1, 2, …) and negative samples having a tendency to have high ranks (…, *N* − 2, *N* − 1, *N*). Therefore, we can ask, What is the probability P(y=1|r) that the item ranked at rank r is in the positive class (y=1)? In this paper, we show that this probability can be mapped to the probability that a fermion (a quantum particle of half-integer spin such as an electron) occupies a given single-particle quantum state in a physical system of independent fermions (28, 29). This probability is known as the Fermi–Dirac (FD) distribution in quantum statistical physics and is used in fields such as atomic physics (30), solid-state physics (31) (e.g., the transport properties of electrons in metals), and astrophysics (32) (e.g., the physics of white dwarf stars).

We explore the application of the FD statistics in machine learning in the context of binary classification problems. Using the FD statistics, we show that the optimal rank threshold below which items are more likely to be positive and above which items are more likely to be negative is the same threshold at which the balanced accuracy of the classifier is maximal and is related to the chemical potential in the FD distribution. We also use the FD distribution to derive a closed-form expression for the variance of the AUC of a classifier, which is independent of the distribution of scores assigned by the classifier. This variance is necessary to assign confidence intervals to the AUC and to estimate sample size in power analysis. Finally, we introduce FiDEL (Fermi–Dirac-based ensemble learning), an ensemble learning algorithm based on the FD distribution that uses the calibrated probability assigned to different base classifiers to combine them into a new ensemble classifier. FiDEL only uses the AUC of the base classifiers and the fraction of positive examples in the problem, both of which can be estimated from the training set.

## The Fermi–Dirac Distribution in Binary Classification

The FD distribution describes the probability that a fermion occupies a single-particle quantum state in a fermionic system, e.g., the probability that an electron occupies a certain atomic level in an atom. Fermions obey the Pauli exclusion principle. This means that if $n_{i}$ represents the number of fermions in quantum state i, then $n_{i}$ can be only 1 or 0. The probability that quantum state i is occupied is then equal to the average occupation number $\left\langle n_{i}\right\rangle$. If the fermionic system is in thermodynamic equilibrium with a thermal bath, then the probability that the quantum state i, assumed to have an energy $\epsilon_{i}$, is occupied follows the FD distribution

$$\left\langle n_{i}\right\rangle =\frac{1}{1+e^{\left(\epsilon_{i}-\mu \right)/k_{B}T}},$$

where $k_{B}$ is the Boltzmann constant, T is the absolute temperature of the thermal bath, and μ is a temperature-dependent chemical potential. The FD distribution can be derived by maximizing the entropy of the system in the microcanonical ensemble of statistical physics under the constraints that the number of fermions $N_{F}$ and the total energy E of the system are known (33):

$$N_{F}=\overset{N_{Q}}{\underset{i=1}{\sum }}\left\langle n_{i}\right\rangle ,$$ [1]

$$E=\overset{N_{Q}}{\underset{i=1}{\sum }}\left\langle n_{i}\right\rangle \epsilon_{i},$$ [2]

where $N_{Q}$ is the number of quantum states available to the fermions. We assume that $N_{Q}$ is finite, which is a good approximation in many physical systems when the energy gap, $\epsilon_{N_{Q}+1}-\epsilon_{N_{Q}}$, $\gg k_{B}T$. For the purpose of this paper quantum states refer to single-fermion quantum states and the $N_{F}$ fermions in our system are noninteracting.

**Fig. 1.**
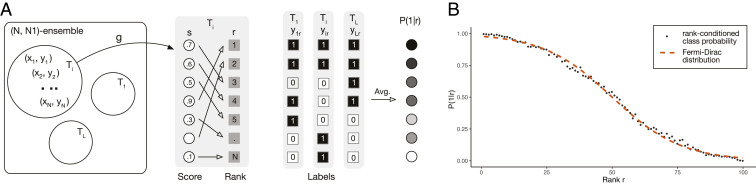
(*A*) Test sets $T_{1},\ldots ,T_{L}$ are sampled from an $\left(N,N_{1}\right)$ ensemble. Each test set consists of N items $x_{i=1}^{N}$ of which exactly $N_{1}$ are in the positive class. Applying the classifier g to set $T_{i}$ endows each item $x_{i}$ with score $s_{i}$. The items are then ranked in decreasing order of scores. If item $x_{i}$ has class y∈{0,1} and was ranked at rank r, then we assign label y to rank r and we keep a tally of the number of times rank r was assigned label y in the L test sets. The rank-conditioned positive class probability P(1|r) is the frequency with which items in the positive class y=1 were ranked at rank r in the L test sets. (*B*) Comparison between the Fermi–Dirac distribution and the rank-conditioned positive class probability for simulated data. An $\left(N=100,N_{1}=50\right)$ ensemble with L=1,000 test sets was simulated. The class-conditioned score density of the classifier was simulated with a Gaussian density function with mean $\mu_{-}=-0.906$ and $\sigma_{-}=1$ for the negative class and $\mu_{+}=0.906$ and $\sigma_{+}=1$ for the positive class. This corresponds to a classifier with an AUC of 0.9. Each test set had 50 items from the positive class and 50 items from the negative class (ρ=1/2). For each of the 1,000 test sets, the items were processed according to *A*. The resulting frequency of positive labels for each rank is plotted and compared with the FD distribution from Eq. **5**, with fitted parameters (β = 0.0759, μ = 50). The Pearson correlation between the FD distribution and the rank-conditioned positive class probability is 0.99 (*P* value < $2.1\times 10^{-124}$).

We next present a conceptual parallel between certain statistical properties in binary classification problems and the FD statistics. Let us define the $\left(N,N_{1}\right)$ ensemble of test sets to be the set (ensemble) of datasets with N items of which exactly $N_{1}$ are in the positive class (for a more formal definition see *SI Appendix*, section 1). Fig. 1*A* depicts test sets in the $\left(N,N_{1}\right)$ ensemble as Venn diagrams with N items each characterized by a feature vector $x_{k}$ and its class $y_{k}$. Let us consider a classifier g that assigns a score s to each item k=1,…,N in one of the test sets $T_{i}$ as shown in Fig. 1*A*. This score is typically a measure of the confidence assigned by classifier g that the item belongs to the positive class and can then be used to rank samples from the most likely to belong to the positive class to the least likely (*SI Appendix*, section 1). The class of the item at rank r can be either $y_{r}=1$ or $y_{r}=0$. The binary nature of the classification of each item suggests that the class of an item at a given rank can be mapped to the binary occupation number of a quantum state in the fermionic system. In this mapping the ranks in the classification problem are the equivalent to the quantum states in the FD problem; class 1 items act as fermions and obey an exclusion principle in that only one item of class 1 can be ranked at any given rank. Classifier g can place either a positive or a negative class item at rank r (Fig. 1*A*). However, for each realization of the test set in the $\left(N,N_{1}\right)$ ensemble, the constraint that $N_{1}=\sum_{r=1}^{N}y_{r}$ must hold. Let us call $\left\langle y_{r}\right\rangle$ the average class of items that the classifier ranked at rank r over all possible test sets in the $\left(N,N_{1}\right)$ ensemble. Given that the previous constraint holds true for each realization, it will also be true on average in the $\left(N,N_{1}\right)$ ensemble; that is,

$$N_{1}=\overset{N}{\underset{r=1}{\sum }}\left\langle y_{r}\right\rangle .$$ [3]

We next discuss the mapping to the classification problem of the energy level $\epsilon_{i}$ of the *i*th quantum state. To do this we note that in any given realization of a test set in the $\left(N,N_{1}\right)$ ensemble, the average rank of positive class samples ${\hat{r}}_{y=1}$ can be expressed as ${\hat{r}}_{y=1}=\sum_{r=1}^{N}ry_{r}/N_{1}$. Calling $\left\langle r|1\right\rangle =\left\langle {\hat{r}}_{y=1}\right\rangle$ the average rank of class 1 items over all possible test sets in the $\left(N,N_{1}\right)$ ensemble, and using that $\left\langle r|1\right\rangle =\left(N+1\right)/2+\left(N-N_{1}\right)\left(1/2-\left\langle AUC\right\rangle \right)$ (*SI Appendix*, *Theorem 1*), where ⟨AUC⟩ is the average AUC of classifier g over the $\left(N,N_{1}\right)$ ensemble, we find that

$$N_{1}\frac{N+1}{2}+N_{1}\left(N-N_{1}\right)\left(\frac{1}{2}-\left\langle AUC\right\rangle \right)=\overset{N}{\underset{r=1}{\sum }}\left\langle y_{r}\right\rangle r.$$ [4]

Comparing Eq. **1** with Eq. **3** and Eq. **2** with Eq. **4** we can postulate a formal mapping of the quantities from the fermionic system to the classification problem: $N_{Q}\to N$, $N_{F}\to N_{1}$, $\left\langle n_{r}\right\rangle \to \left\langle y_{r}\right\rangle$, $\epsilon_{r}\to r$, and $E\to N_{1}\left(N+1\right)/2+N_{1}\left(N-N_{1}\right)\left(1/2-\left\langle AUC\right\rangle \right)$. Given that $y_{r}$ takes only the values 0 and 1, its ensemble average $\left\langle y_{r}\right\rangle$ is equal to the probability P(y=1|r) that an item is in the positive class given that it was ranked at rank r. Under these conditions and from the fact that the FD distribution follows from the second principle of thermodynamics along with Jaynes’ insight (34) that the maximum-entropy principle in statistical mechanics is nothing but the maximization of the uncertainty about our unknowns, we conclude the maximum-entropy rank-conditioned class probability in the classification problem is given by the FD distribution with the appropriately mapped quantities:

$$P\left(y=1|r\right)=\frac{1}{1+e^{\beta \left(r-\mu \right)}},$$ [5]

where β and μ are chosen to fit Eqs. **3** and **4** from the known $N_{1}$ and ⟨AUC⟩ of the classifier. Fig. 1*B* shows the result of plotting $\left\langle y_{r}\right\rangle$ (dots) for an $\left(N,N_{1}\right)$ ensemble with N=100 and $N_{1}=50$ and an ⟨AUC⟩ of 0.9 and the fitted FD distribution (red dashed line), which follows the empirically simulated distribution remarkably well (*P* value < $2.1\times 10^{-124}$).

To recap, the FD distribution for a physical system follows from the second principle of thermodynamics (maximum entropy) under the constraints that the energy and the number of fermions of the system are known. Because of the mapping between the binary classification problem and the fermionic system, we can think of the FD distribution as the maximum-entropy estimate of the rank-conditioned class probability with the appropriately mapped constraints. The rank-conditioned class probability can also be derived directly from these constraints and the maximum-entropy principle without invoking a mapping between the classification problem and the fermionic system (*SI Appendix*). However, we believe that this mapping can provide a fruitful analogy to interpret the parameters β and μ, as we will see in the next section.

It should be clear from this discussion that we are not claiming that the rank-conditioned class probability is the FD distribution. Rather, we claim that the FD distribution is the distribution that makes the least number of assumptions by maximizing our uncertainty about the information we do not have but taking into account the information encoded in the aforementioned constraints. If more information were available, for example, if we knew the class-conditioned score density of the classifier, then a more precise distribution could be derived. However, the FD distribution provides an excellent approximation for the posterior probability of binary classifiers as is shown in the following sections where we introduce multiple applications of this approach.

## The Temperature and Chemical Potential in Binary Classification

Next, we discuss the interpretation of the temperature and the chemical potential in the context of binary classification. In a fermionic system, as the temperature approaches 0, all fermions will occupy the quantum states with the lowest possible energies allowed by the exclusion principle up to the chemical potential at T=0, a quantity known as the Fermi energy $\epsilon_{F}$. On the other temperature extreme, when T→∞, all quantum states are equally probable and the average occupation number is $N_{F}/N_{Q}$. In the classification problem, the parameter β is mapped to the inverse temperature in the physical system. As β→∞ the FD distribution is a step function and is equal to 1 for ranks less than or equal to $N_{1}$ and 0 otherwise. This corresponds to a perfect classifier with an ⟨AUC⟩ of 1. Note that, in this case, the chemical potential μ is equal to $N_{1}$. When β decreases (i.e., the temperature increases), the probability P(y=1|r) that an item is of class 1 at rank r becomes a smooth logistic function, which reflects an imperfect classification with an ⟨AUC⟩ between 0.5 and 1. For β→0 (i.e., T→∞), $P\left(y=1|r\right)\to N_{1}/N$ independently of r, which corresponds to a random classifier. The above discussion suggests that the temperature in a fermionic system maps to classification errors. At finite temperature there is no clear-cut energy threshold below which energy states are occupied by fermions and above which the states are unoccupied. In the classification problem, that means that we do not have a clear-cut threshold rank below which we will find only class 1 items and above which we will find only class 0 items. We show later that at finite temperature the optimal threshold in the classification problem is related to the chemical potential in the physical system.

**Fig. 2.**
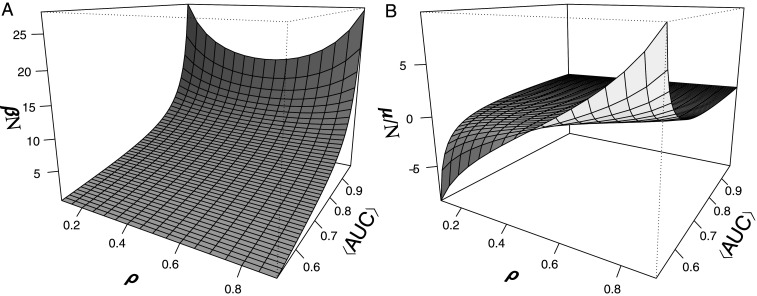
The rescaled coefficients of the Fermi–Dirac distribution are determined from the values of ρ and ⟨AUC⟩. (*A*) Dependence of βN on ⟨AUC⟩ and the prevalence, ρ. (*B*) Dependence of μ/N on ⟨AUC⟩ and the prevalence ρ. Here, β and μ were calculated as discussed in *SI Appendix* with N=1,000.

The parameters β and μ should be computed from the constraints Eqs. **3** and **4** and in general will depend on N, $N_{1}$, and ⟨AUC⟩. However, for sufficiently large N, these parameters can be rescaled such that βN and μ/N can be computed numerically from the knowledge of ρ and ⟨AUC⟩ only (*SI Appendix*, section 5), where $\rho =N_{1}/N$ is the fraction of class 1 items, often called prevalence. Fig. 2 shows the dependence of βN (Fig. 2*A*) and μ/N (Fig. 2*B*) as a function of the ⟨AUC⟩ and ρ. While a general analytical expression to express βN and μ/N in terms of ρ and ⟨AUC⟩ does not exist, it is possible to express μ as a function of β, N, and $N_{1}$:

$$\frac{\mu }{N}=\frac{1}{2}-\frac{1}{\beta N}\ln\left[\frac{\sinh\left(\beta N\left(1-\rho \right)/2\right)}{\sinh\left(\beta N\rho /2\right)}\right].$$ [6]

It is also possible to find explicit expressions for special cases. For weak classifiers, i.e., when (⟨AUC⟩→0.5), we show in *SI Appendix* that

$$\begin{array}{ll} \beta N & =12\left(\left\langle AUC\right\rangle -0.5\right),\\ \frac{\mu }{N} & =\frac{1}{2}-\frac{1}{12\left(\left\langle AUC\right\rangle -0.5\right)}\ln\left(\frac{1-\rho }{\rho }\right). \end{array}$$

Another approximate expression can be found in the limit of perfect classifiers $\left(\left\langle AUC\right\rangle \to 1^{-}\right)$ (*SI Appendix*),

$$\begin{array}{ll} \beta N & =\sqrt{\frac{2}{3}}\frac{1}{\sqrt{\rho \left(1-\rho \right)\left(1-\left\langle AUC\right\rangle \right)}},\\ \frac{\mu }{N} & =\rho . \end{array}$$

Finally, if ρ=1/2, then μ=N/2 for all ⟨AUC⟩ (*SI Appendix*). Beyond the special cases discussed above, there are symmetries in the dependence of βN and μ/N as a function of ⟨AUC⟩ and ρ that must hold for all 0≤⟨AUC⟩≤1 and 0≤ρ≤1, as discussed in *SI Appendix*.

## Choosing Thresholds in Binary Classification

To assign class labels to each sample in a test set we must choose a decision threshold. In practical applications, this threshold is typically learned from a training set. But, if we know the rank-conditioned class probability, it is possible to relate the rank-threshold $r^{*}$ below/above which the classes are assigned to be positive/negative to the parameters β and μ of the corresponding FD distribution.

This threshold can be chosen to be the rank at which the class-conditioned rank probability that an item is at rank r is the same for the positive and negative classes. We define the log-likelihood ratio as

$$L\left(r\right)=\ln\left(\frac{P\left(r|1\right)}{P\left(r|0\right)}\right)=\ln\left(\frac{P\left(1|r\right)}{P\left(0|r\right)}\frac{1-\rho }{\rho }\right),$$ [7]

where in the second equality we applied Bayes’ theorem to express the class-conditioned rank probability in terms of the posterior rank-conditioned class probability P(r|y)=P(y|r)P(r)/P(y) and used that P(y=1)=ρ. Using Eq. **5** as the rank-conditioned class probability we can find that

$$L\left(r\right)=\ln\frac{1-\rho }{\rho }-\beta \left(r-\mu \right).$$ [8]

Hence, the optimal rank threshold can be computed as the rank that makes $L\left(r^{*}\right)=0$:

$$\frac{r^{*}}{N}=\frac{\mu }{N}+\frac{1}{\beta N}\ln\frac{1-\rho }{\rho }$$ [9]

$$=\frac{1}{2}+\frac{1}{\beta N}\ln\left[\frac{1-\rho }{\rho }\frac{\sinh\left(\beta N\rho /2\right)}{\sinh\left(\beta N\left(1-\rho \right)/2\right)}\right],$$ [10]

where we used Eq. **6** to go from Eq. **9** to Eq. **10**. Eq. **10** shows the dependence of the optimal threshold on β. From the previous section, this means that the only information needed to determine the optimal threshold is the ⟨AUC⟩ and the prevalence ρ, which can be learned from the training set.

It is also possible to find a threshold that strikes a compromise between the sensitivity and the specificity of a classifier. For a ranked list, a popular way to do this is to find the rank r that maximizes the balanced accuracy bac(r), defined as the average of the true positive rate TPR(r) and the specificity or 1 − FPR(r) (where FPR denotes the false positive rate) of a binary classifier. For a given instance of the test set, these metrics can be expressed as

$$\text{TPR}\left(r\right)=\frac{1}{N_{1}}\overset{r}{\underset{i=1}{\sum }}y_{i},$$ [11]

$$1-\text{FPR}\left(r\right)=\frac{1}{N-N1}\overset{N}{\underset{i=r+1}{\sum }}\left(1-y_{i}\right),$$ [12]

$$\text{bac}\left(r\right)=\frac{1}{2}\left(\text{TPR}\left(r\right)+1-\text{FPR}\left(r\right)\right).$$ [13]

Taking the average of the previous equations in the $\left(N,N_{1}\right)$ ensemble we can express the average balanced accuracy in terms of the posterior class distribution as

$$\left\langle \text{bac}\left(r\right)\right\rangle =\frac{1}{N_{1}}\overset{r}{\underset{i=1}{\sum }}P\left(1|i\right)+\frac{1}{N-N1}\overset{N}{\underset{i=r+1}{\sum }}P\left(0|i\right).$$ [14]

The next step in finding the optimal threshold is to choose the argument r that maximizes ⟨bac(r)⟩. We approximate this step by assuming r to be a continuous variable and finding the value of r that makes the derivative of ⟨bac(r)⟩ zero; that is,

$$\frac{d\;\left\langle \text{bac}\left(r\right)\right\rangle }{dr}{|}_{r=r^{*}}=0.$$ [15]

Assuming N≫1 so that that the discrete sums in Eqs. **11** and **12** can be approximated by integrals, we find that Eq. **15** yields

$$\frac{P\left(1|r^{*}\right)}{P\left(0|r^{*}\right)}=\frac{\rho }{1-\rho }.$$ [16]

To ascertain that the $r^{*}$ resulting from Eq. **15** is a maximum, we need to verify that the second derivative of the ⟨bac(r)⟩ is negative at $r^{*}$. Using the FD expression for the distribution of P(y|r) we find that $\frac{d^{2}\left\langle \text{bac}\left(r\right)\right\rangle }{dr^{2}}{|}_{r=r^{*}}=-\beta$, which is always negative when the classifier is better than random; i.e., ⟨AUC⟩>1/2, as β is positive in those cases (Fig. 2). Interestingly, Eq. **16** yields the same result as the earlier calculation requiring that the log-odds ratio $L\left(r^{*}\right)=0$. In other words, the threshold $r^{*}$ that makes the log ratio of class-conditioned rank distributions zero is also the one that maximizes the balanced accuracy.

**Fig. 3.**
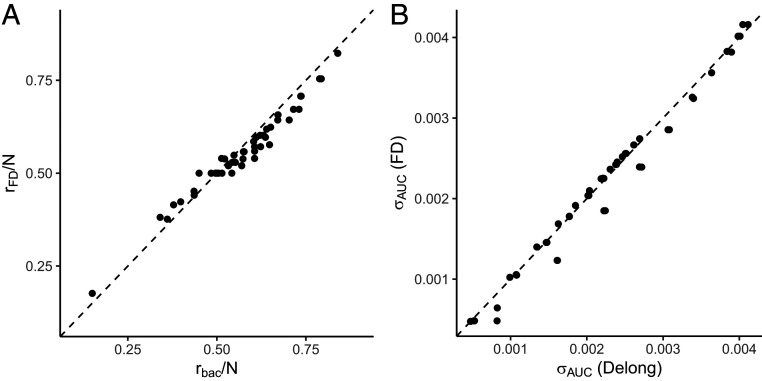
(*A*) Correlation between two different methods for determining the optimal thresholds for segregating positive and negative classes. $r_{\text{bac}}$ is the traditional method of scanning over all possible rank thresholds to empirically determine the rank that maximizes the balance accuracy and $r_{FD}$ is the proposed closed-form method, Eq. **9**, based on the FD distribution. Here, nine different ρ values ranging from 0.1 to 0.9 and five different ⟨AUC⟩ values ranging from 0.55 to 0.95 were tested with total sample size N=50,000. The correlation coefficient is R=0.98 (*P* value $<2\times 10^{-16}$). (*B*) Correlation between two different methods for determining the SD of the AUC. $\sigma_{\text{AUC}}$ (DeLong) represents the DeLong method, and $\sigma_{\text{AUC}}$ (FD) represents the FD-based method. The same conditions as in *A* were used. The correlation coefficient is R=0.99 (*P* value $<2\times 10^{-16}$).

To exemplify and verify the calculations described in this section we use simulation experiments based on classifiers with Gaussian class-conditioned score densities. The simulations consist of 45 realizations of test sets with N=50,000, 0.55≤⟨AUC⟩≤0.95, and 0.1≤ρ≤0.9. Fig. 3*A*, in which each point represents a different combination of ρ and ⟨AUC⟩, shows that the threshold $r_{FD}$ calculated using Eq. **9** based on the requirement that $L\left(r^{*}\right)=0$ is an excellent approximation of the threshold $r_{bac}$ [computed by finding the r that maximizes the ⟨bac(r)⟩ from scanning through all possible thresholds for each realization of the test set in the simulations]. The actual r that maximizes the balanced accuracy and the estimate using the FD expression have a correlation coefficient R=0.98. The probability that such a high correlation (or larger) between the two quantities is due to chance is negligible (*P* value < $2\times 10^{-16}$).

## The Variance of AUC Estimates

The AUC is perhaps the most popular metric to evaluate the performance of binary classifiers. While it would be desirable to know the distribution of the AUC of a given classifier over all possible test sets with similar characteristics [such as the $\left(N,N_{1}\right)$ ensemble of test sets we discussed earlier], what we usually compute in most applications is an estimator of its mean value ⟨AUC⟩ in a specific dataset. This estimate of the AUC carries an error that results from inevitable sample-to-sample variation and finite sample sizes. Therefore, any complete reporting of the AUC should also provide a confidence interval that contains the true but unknown ⟨AUC⟩ with some probability, typically 95%. To compute this confidence interval it is necessary to estimate the variance $\sigma_{\text{AUC}}^{2}$ of the AUC distribution. As discussed in refs. 27 and 35, the mean and variance of the AUC distribution for a classifier are given by

$$\left\langle AUC\right\rangle =\text{Prob}\left(s_{i}>s_{j}|y_{i}=1;y_{j}=0\right),$$ [17]

$$\begin{array}{ll} \sigma_{AUC}^{2} & =\frac{1}{N_{1}N_{0}}\left[\left\langle AUC\right\rangle \left(1-\left\langle AUC\right\rangle \right)\right.\\ & +\left(N_{1}-1\right)\left(P_{110}-{\left\langle AUC\right\rangle }^{2}\right)\\ & \left.+\left(N_{0}-1\right)\left(P_{100}-{\left\langle AUC\right\rangle }^{2}\right)\right], \end{array}$$ [18]

where $N_{0}=N-N_{1}$, $P_{110}=\text{Prob}\left(\min\left(s_{i},s_{j}\right)>s_{k}|y_{i}=y_{j}=1;y_{k}=0\right)$ is the probability that the classifier assigns higher scores to two randomly and independently sampled positive items than to a randomly sampled negative item, and $P_{100}=\text{Prob}\left(s_{i}>\max\left(s_{j},s_{k}\right)>s_{k}|y_{i}=1,y_{j}=y_{k}=0\right)$ is the probability that the classifier assigns lower scores to two randomly and independently sampled negative items than to a randomly sampled positive item. We also denote by $P_{10}$ the probability $\text{Prob}\left(s_{i}>s_{j}|y_{i}=1;y_{j}=0\right)$ that the classifier assigns a higher score to a randomly sampled positive item than to a randomly sampled negative item.

We derive Eqs. **17** and **18** in *SI Appendix*, but for completeness we provide some intuition for these formulas here. The AUC of a classifier measures the area under the receiver operating characteristic (ROC) curve traced by the points (FPR(s), TPR(s)) for a given test set, where the parameter s is the classification threshold discussed in the previous section and ranges from the maximum to the minimum possible scores outputted by the classifier. When s is the classification threshold, all the items with scores larger than s are considered positives, so the TPR(s) is the fraction of the $N_{1}$ positive items with scores larger than s and the FPR(s) is the fraction of the $N_{0}$ negative items with scores larger than s. When we compute the area under the ROC curve using a rectangular integration rule, each time the parameter s crosses the score of a positive example, the TPR gains $1/N_{1}$ units whereas the FPR does not change. This corresponds to a vertical change in the ROC curve and therefore there is no gain in AUC. When the score s crosses the value $s_{k}$ of a negative item k ($k=1,\ldots ,N_{0}$), the ROC curve goes from point (FPR$\left(s_{k-1}\right)$, TPR$\left(s_{k}\right)$) to point (FPR$\left(s_{k}\right)$, TPR$\left(s_{k}\right)$), with FPR$\left(s_{k}\right)$ = FPR$\left(s_{k-1}\right)+1/N_{0}$. The AUC results from adding the areas of $N_{0}$ rectangles (one per negative item k with score $s_{k}$) whose height is equal to the fraction of positive examples TPR $\left(s_{k}\right)$ with score larger than $s_{k}$ and whose base is equal to $1/N_{0}$, which is the x-axis change in the ROC curve that takes place when the parameter s goes from one negative item to the next. Interestingly, the calculation sketched above is the exact same calculation that we would perform to estimate the frequency with which we find positive items with score larger than that of a negative example in the same test set: Given a negative item k for which the classifier assigned a score $s_{k}$, the frequency of positive examples with scores greater than $s_{k}$ coincides with the TPR $\left(s_{k}\right)$; the probability of a positive to have a score greater than a negative is the sum of these frequencies weighted by the probability of choosing that negative sample, which in a given test set is $1/N_{0}$. We have just justified that in a given test set and for a given classifier, the AUC can be computed as

$$AUC=\frac{1}{N_{0}}\overset{N_{0}}{\underset{k=1}{\sum }}\frac{1}{N_{1}}\overset{N_{1}}{\underset{i=1}{\sum }}H\left(s_{P,i}-s_{N,k}\right),$$ [19]

where $s_{P,i}$ and $s_{N,k}$ are the scores assigned by the classifier to the ith positive examples and the kth negative examples, respectively, and H(s) is the Heaviside function that takes the value of 1 for positive arguments and 0 for negative arguments. Eq. **19** expresses a known relation between the AUC of a classifier in a given test set and the Mann–Whitney statistics $U=\sum_{k=1}^{N_{0}}\sum_{i=1}^{N_{1}}H\left(s_{P,i}-s_{N,k}\right)$ (36). Taking the expected value in both sides of the equality in Eq. **19** we get $\left\langle AUC\right\rangle =\left\langle H\left(s_{P}-s_{N}\right)\right\rangle$. Note that the expected value of $H\left(s_{P}-s_{N}\right)$ for randomly and independently sampled positive and negative examples with scores $s_{P}$ and $s_{N}$, respectively, is equal to the probability that a positive example has a score larger than a negative example; that is, $\left\langle H\left(s_{P}-s_{N}\right)\right\rangle =\text{Prob}\left(s_{i}>s_{j}|y_{i}=1;y_{j}=0\right)$. Therefore, $\left\langle AUC\right\rangle =\text{Prob}\left(s_{i}>s_{j}|y_{i}=1;y_{j}=0\right)$, which proves Eq. **17**. (For an alternative derivation see *SI Appendix*.)

Next, we sketch the derivation of Eq. **18**, which will allow us to elucidate the origin of the parameters $P_{110}$ and $P_{100}$. (See *SI Appendix* for the full derivation.) To compute the variance of the AUC we use the fact that $\sigma_{AUC}^{2}=\left\langle AUC^{2}\right\rangle -{\left\langle AUC\right\rangle }^{2}$, which requires squaring Eq. **19** and taking its expected value in the $\left(N,N_{1}\right)$ ensemble. This operation leads to four nested sums (two over the positive examples and two over the negative examples) of the average of $H\left(s_{P,i}-s_{N,k}\right)H\left(s_{P,j}-s_{N,m}\right)$. To deal with repeated indexes in these nested sums we consider the following four cases: 1) Case i≠j and k≠m leads to $N_{0}\left(N_{0}-1\right)N_{1}\left(N_{1}-1\right)$ terms of the form $\left\langle H\left(s_{P,i}-s_{N,k}\right)H\left(s_{P,j}-s_{N,m}\right)\right\rangle$, all of which are equal to ${\left\langle AUC\right\rangle }^{2}$, given that $H\left(s_{P,i}-s_{N,k}\right)$ and $H\left(s_{P,j}-s_{N,m}\right)$ are independent (because $s_{P,i}$, $s_{N,k}$, $s_{P,j}$, and $s_{N,m}$ are), and $\left\langle H\left(s_{P,i}-s_{N,k}\right)\right\rangle =\left\langle H\left(s_{P,j}-s_{N,m}\right)\right\rangle =\left\langle AUC\right\rangle$. 2) Case i=j and k≠m leads to $N_{0}\left(N_{0}-1\right)N_{1}$ terms of the form $\left\langle H\left(s_{P,i}-s_{N,k}\right)H\left(s_{P,i}-s_{N,m}\right)\right\rangle$, which is equal to the probability earlier denoted by $P_{100}$ that the score of a randomly sampled positive item ($s_{P,i}$) is larger than the scores of two independently and randomly sampled negative items ($s_{N,k}$ and $s_{N,m}$). 3) Case i≠j and k=m leads to $N_{0}N_{1}\left(N_{1}-1\right)$ terms of the form $\left\langle H\left(s_{P,i}-s_{N,k}\right)H\left(s_{P,j}-s_{N,k}\right)\right\rangle$, which is equal to the probability earlier denoted by $P_{110}$ that both the scores of two independently and randomly sampled positive items ($s_{P,i}$ and $s_{P,j}$) are larger than the scores of randomly sampled negative items ($s_{N,k}$). 4) Case i=j and k=m leads to $N_{0}N_{1}$ terms of the form $\left\langle H{\left(s_{P,i}-s_{N,k}\right)}^{2}\right\rangle$, which, given that $H{\left(s\right)}^{2}=H\left(s\right)$, is equal to the probability that the score of a randomly sampled positive item is larger than the score of a randomly sampled negative item, which was shown before to be equal to ⟨AUC⟩. Assembling all these cases to compute $\sigma_{AUC}^{2}$, we recover Eq. **18**.

Using Eq. **18** requires knowledge of the quantities $P_{110}$ and $P_{100}$ that depend on the generally unknown class-conditioned score densities P(s|y). One could proceed by assuming some functional form for these densities. For example, if P(s|y) is assumed to be exponential, it can be shown (27) that $P_{110}=AUC/\left(2-AUC\right)$ and $P_{100}=2AUC^{2}/\left(1+AUC\right)$. However, assuming a distribution just because it yields analytical expressions may lead to inaccurate results, e.g., producing too loose confidence intervals. In practical applications the variance of the AUC is often computed using a method first proposed by DeLong et al. (37), which consists of rearranging the terms in Eq. **19** into an estimator of the AUC variance,

$$\begin{array}{ll} & \sigma_{AUC}^{2}\left(\text{DeLong}\right)=\\ & \frac{1}{N_{1}\left(N_{1}-1\right)}\overset{N_{1}}{\underset{i=1}{\sum }}{\left[\frac{1}{N_{0}}\overset{N_{0}}{\underset{j=1}{\sum }}H\left(s_{P,i}-s_{N,j}\right)-AUC\right]}^{2}+\\ & \frac{1}{N_{0}\left(N_{0}-1\right)}\overset{N_{0}}{\underset{j=1}{\sum }}{\left[\frac{1}{N_{1}}\overset{N_{1}}{\underset{i=1}{\sum }}H\left(s_{P,i}-s_{N,j}\right)-AUC\right]}^{2}. \end{array}$$ [20]

Eq. **20** has proved to be a reliable option for the computation of $\sigma_{AUC}^{2}$ (38, 39).

Note that $P_{10}$, $P_{110}$, and $P_{100}$ depend only on the relative order of positive and negative samples. As such, they could be written in terms of the class-conditioned rank probabilities that in turn can be expressed using the FD distribution for the rank-conditioned class probabilities. Indeed, in cases where we do not know the true class-conditioned score or rank distribution, Eq. **18** requires that we use only $N_{0}$, $N_{1}$, and ⟨AUC⟩ and assume the most parsimonious (maximum-entropy) distribution for the rank-conditioned class probability. As was shown earlier, this leads to the FD distribution Eq. **5**.

Let us first express $P_{10}$ (i.e., the right-hand side of Eq. **17**) in terms of ranks. The probability that the score of a negative item is smaller than the score of a positive item translates into the probability that the negative item has a higher rank than that of a positive item. If a positive item is at rank r, the probability that a negative item has a rank higher than r is $\sum_{i=r+1}^{N}P\left(i|0\right)$. As the positive item can be at any rank r, to compute $P_{10}$ we need to add the previous sum over all the possible ranks where the positive item is, weighted by the probability P(r|1) that there is a positive item at rank r. Using that $P\left(r|1\right)=P\left(1|r\right)/N_{1}$ and $P\left(r|0\right)=P\left(0|r\right)/N_{0}$, Eq. **17** can be expressed as

$$\begin{array}{ll} \left\langle AUC\right\rangle & =\frac{1}{N_{1}N_{0}}\overset{N}{\underset{r=1}{\sum }}\overset{N}{\underset{i=r+1}{\sum }}P\left(1|r\right)P\left(0|i\right)\\ & =\frac{1}{N_{1}N_{0}}\overset{N}{\underset{r=1}{\sum }}\overset{N}{\underset{i=r+1}{\sum }}\frac{1}{1+e^{\beta \left(r-\mu \right)}}\frac{e^{\beta \left(i-\mu \right)}}{1+e^{\beta \left(i-\mu \right)}}, \end{array}$$ [21]

where we expressed P(1|r) in terms of the FD distribution. Recall that β and μ were selected using constraints based on the number of positive samples (Eq. **3**) and the ⟨AUC⟩ (Eq. **4**). These constraints are different from Eq. **21**, and therefore Eq. **21** may appear to overdetermine the parameters. Interestingly this is not the case. We show in *SI Appendix* that Eq. **21** holds for any rank-conditioned class probability P(y|r) that verifies those two constraints, and therefore they are valid for the FD distribution whose parameters were fitted using those very same conditions.

Following similar arguments to the ones used to deduce Eq. **21**, we can find expressions for $P_{110}$ and $P_{100}$:

$$P_{110}=\frac{1}{N_{1}^{2}N_{0}}\overset{N}{\underset{i=1}{\sum }}\overset{N}{\underset{j=1\neq i}{\sum }}\overset{N}{\underset{r=\max\left(i,j\right)+1}{\sum }}P\left(1|i\right)P\left(1|j\right)P\left(0|r\right)$$ [22]

$$P_{100}=\frac{1}{N_{1}N_{0}^{2}}\overset{N}{\underset{i=1}{\sum }}\overset{N}{\underset{j=1\neq i}{\sum }}\overset{\min\left(i,j\right)-1}{\underset{r=1}{\sum }}P\left(1|r\right)P\left(0|i\right)P\left(0|j\right),$$ [23]

where $P\left(1|r\right)=\frac{1}{1+e^{\beta \left(r-\mu \right)}}$ and $P\left(0|r\right)=\frac{e^{\beta \left(r-\mu \right)}}{1+e^{\beta \left(r-\mu \right)}}$.

We compare the SD $\sigma_{AUC}$ estimated using Eq. **20** [DeLong et al.’s (37) method] and using Eq. **18** (with $P_{110}$ and $P_{100}$ computed using the FD-based method of Eqs. **22** and **23**) in Fig. 3*B* for the same simulations as those used in Fig. 3*A*. The two ways of computing the SD yield almost identical values, with a correlation coefficient R=0.99 (*P* value < $2\times 10^{-16}$). The minor deviations between the two ways of computing $\sigma_{AUC}$ observed in Fig. 3 correspond to cases where the prevalence ρ takes values close to 0 or 1. In these situations, the FD distribution fit to the constraints is not as good compared to cases where the prevalence is of intermediate value.

## Using the FD Distribution for Ensemble Classification

Ensemble learning for classification is the endeavor of combining multiple base classifiers in an effort to construct an ensemble classifier that generalizes better than any of its constituents. In this section, we present FiDEL, an ensemble learning method based on using the FD distribution to model the rank-conditioned class probabilities for different base classifiers.

We assume that we have M classifiers in the ensemble, denoted by $g_{i=1}^{M}$. Let $r_{ik}$ denote the rank assigned to item k by classifier i. Let $P\left(r_{1k},r_{2k},\ldots ,r_{Mk}|y\right)$ denote the joint probability of rank assignment by classifiers $g_{i=1}^{M}$ given the class y∈{0,1} of item k. Following refs. 22 and 23, we assume that the base classifiers’ rank assignments for a given item are conditionally independent given the class. This strong assumption means that different classifiers rank the same item independently of each other whether the item is in the positive or the negative class. Under this assumption, the joint class-conditional distribution of rank predictions can be written as

$$P\left(r_{1k},r_{2k},\ldots ,r_{Mk}|y\right)=P\left(r_{1k}|y\right)\ldots P\left(r_{Mk}|y\right).$$ [24]

We use the log-likelihood ratio

$$L_{\text{FiDEL}}\left(k\right)=\ln\left(\frac{P\left(r_{1k},r_{2k},\ldots ,r_{Mk}|1\right)}{P\left(r_{1k},r_{2k},\ldots ,r_{Mk}|0\right)}\right),$$

to estimate the degree to which the evidence given by the ranks assigned by classifiers to item k supports the conclusion that item k is in the positive versus the negative class. Using the assumption of conditional independence, the log-likelihood ratio can be rewritten as

$$L_{\text{FiDEL}}\left(k\right)=\ln\left(\frac{P_{1}\left(r_{1k}|1\right)}{P_{1}\left(r_{1k}|0\right)}\right)+\cdots +\ln\left(\frac{P_{M}\left(r_{Mk}|1\right)}{P_{M}\left(r_{Mk}|0\right)}\right),$$ [25]

where $P_{i}$ is the probability of rank given class for classifier $g_{i}$.

Replacing $P_{i}\left(r|y\right)$ by the FD distribution, the sum in the right-hand side of Eq. **25** can be expressed as

$$L_{\text{FiDEL}}\left(k\right)=\overset{M}{\underset{i=1}{\sum }}\beta_{i}\left(r_{i}^{*}-r_{ik}\right),$$ [26]

where

$$r_{i}^{*}=\mu_{i}+\frac{1}{\beta_{i}}\ln\frac{1-\rho }{\rho }.$$

$L_{\text{FiDEL}}\left(k\right)$ can be used as the score provided by the FiDEL ensemble classifier to rank items to compute the AUC. Items that get larger and positive $L_{\text{FiDEL}}$ scores will be more likely to belong to the positive class. Conversely, more negative scores will be more likely to belong to the negative class. The log-likelihood ratio suggests that 0 is the natural threshold that separates items in the positive and negative classes, and therefore the predicted label for the FiDEL ensemble is

$$y_{k}^{\text{FiDEL}}=H\left\{L_{\text{FiDEL}}\left(k\right)\right\},$$

where H is the Heaviside step function.

Note that the contribution to $L_{\text{FiDEL}}$ of classifier i is the difference between the optimal threshold $r_{i}^{*}$ and the rank r of the item being classified, weighted by the parameter $\beta_{i}$. As previously discussed, β can be interpreted as the inverse of the temperature of an equivalent physical system; a higher temperature corresponds to more classification errors and thus lower accuracy. (See Fig. 2*A* where it can be seen that for any ρ, β increases monotonically with the ⟨AUC⟩ of a classifier.) Therefore, weighting each classifier’s contribution to $L_{\text{FiDEL}}$ by β can be easily interpreted: Methods with higher error map to higher temperatures that lead to lower βs, which results in a lower weight in the final score. The predicted score of the ensemble classifier, $L_{\text{FiDEL}}$, is also dependent on the rank $r_{ik}$ assigned by the classifier i relative to the threshold $r_{i}^{*}$. Items ranked lower and farther from the threshold $r_{i}^{*}$ given by classifier i will contribute to a larger $L_{\text{FiDEL}}$.

The derivation of the FiDEL ensemble is based on the strong assumption that base classifier predictions are class-conditionally independent. To determine the extent to which class-conditional dependence influences the performance of the FiDEL ensemble we developed a model (*SI Appendix*) that simulates the situation in which all pairs of classifiers in the ensemble have a conditional rank correlation given both the positive and the negative class equal to a parameter $\hat{r}$, which we varied between 0 (uncorrelated case) and 0.6. In practice, however, there are different degrees of correlation between different pairs of classifiers, as we will see below. For different values of the class-conditioned rank correlation $\hat{r}$ we compared the performance of FiDEL with that of the best classifier in the ensemble and with a baseline ensemble model that we call the wisdom of crowds (WoC) ensemble (40). The WoC ensemble is a classifier whose score for a given item can be computed as the average of the ranks assigned by the base classifiers to that item. The results of these simulations, summarized in *SI Appendix*, Figs. S1 and S2, show that the performance of FiDEL is robust to mild violations in the assumption of class-conditional independence and that FiDEL’s performance is greater than that of the best individual classifiers up to a class-conditioned correlation of $\hat{r}≲$ 0.4. Furthermore, FiDEL is better than the WoC ensemble for all values of $\hat{r}$ tested. To exemplify its use and assess its performance in practical classification tasks, we applied FiDEL to two problems proposed in the Kaggle crowd-sourcing platform: the West Nile Virus (WNV) Prediction challenge (41) and the Springleaf Marketing Response (SMR) challenge (42) (*Materials and Methods* and *SI Appendix*, Table S1). We chose these challenges because they are binary classification problems with vastly different positive-class prevalence (ρ=0.08 and 0.24 for the WNV and SMR challenges, respectively) and large datasets (N=10,506 and 22,000 points for the WNV and SMR challenges, respectively) and the data are easily accessible though the Kaggle website. We used 23 general purpose and widely used methods as base classifiers, of which 21 were used in the WNV data and 20 were used in the SMR challenge (we intended to also use 21 classifiers in the SMR data, but one of the chosen classifiers failed to run; *SI Appendix*, Table S2). In both problems, the data were randomly partitioned into 22 equal-size subgroups, each of which maintained the class proportions of the overall dataset. Of these 22 groups, 21 were used for training and validation and the remaining one was used as the test set.

As discussed above, a high degree of class-conditioned correlation can considerably degrade FiDEL’s performance. We studied the class-conditioned correlation under two different training strategies. In the “disjoint partition” strategy, we trained each of the classifiers in its own partition, in such a way that no classifier was trained using the same data. In the “same partition” strategy, all classifiers were trained using the same partition. The class-conditioned correlation averaged over the two classes for each pair of classifiers for the prediction in the test set in both the WNV and the SMR datasets is shown in *SI Appendix*, Fig. S3. The average class-conditioned correlations $\hat{r}$ over all the pairs of classifiers in the WNV data for the same partition and the disjoint partition strategies were 0.66 and 0.54. For the SMR data the correlations $\hat{r}$ for the same partition and the disjoint partition were 0.44 and 0.32. These results suggest that the best strategy to use FiDEL from the perspective of minimizing the class-conditioned correlation is the disjoint partition strategy, which we use next.

**Fig. 4.**
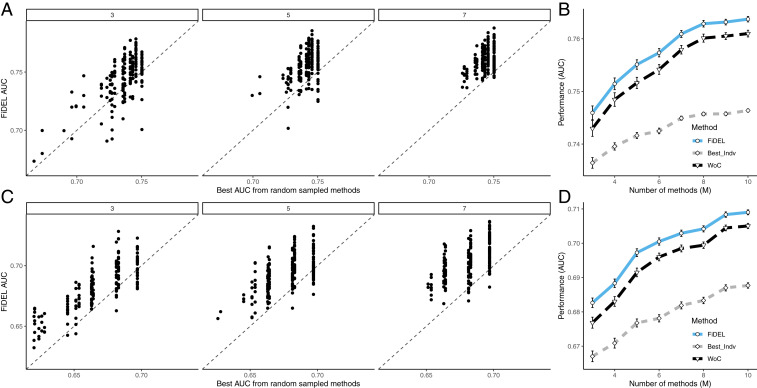
Performance of FiDEL on two Kaggle binary classification challenges: the WNV Prediction challenge (*A* and *B*) and the SMR challenge (*C* and *D*). The dataset in the WNV Prediction challenge has a prevalence ρ=0.08 and 10,506 data points. The dataset in the SMR challenge has a prevalence ρ=0.24 and 22,000 data points. (*A* and *C*) Comparison between the AUCs using FiDEL by combining M algorithms randomly chosen among 21 (WNV) or 20 (SMR) possible algorithms (y axis) and the AUC of the best among the M algorithms used in the combination (x axis) with M=3, 5, and 7. Each point corresponds to one of 200 combinations of randomly chosen classifiers. (*B* and *D*) The average and SEM of the AUCs over 200 combinations of M algorithms (M=3,…,10) chosen randomly among 21 (WNV) or 20 (SMR) methods for FiDEL (blue solid line), WoC (black dashed line), and the best individual algorithm among the M combined (gray dashed line) for the WNV Prediction (*B*) and SMR (*D*) challenges. FiDEL was trained using the AUC and ρ values of the base classifiers using a validation set carved from the training set. The AUCs reported here correspond to evaluations of base classifiers, FiDEL, and WoC in the test set, which is a partition independent of the training set. Different partition choices and base classifier combinations may produce slightly different results.

After training in their respective training set, the $AUC_{i}$ of each classifier i and the prevalence $\rho_{i}$ were computed in the remaining training partitions (that is, excluding partition i and the test set). $AUC_{i}$ and $\rho_{i}$ were then used to fit the parameters $\beta_{i}$ and $\mu_{i}$ of the FD distribution for each classifier. *SI Appendix*, Fig. S4 shows that the resulting FD distribution with the fitted β and μ is an excellent approximation to the empirically computed rank-conditioned class probability. We then applied the learned FiDEL ensemble method to the test data (Fig. 4). We compared the performance of FiDEL to that of the WoC ensemble method and the best individual classifier. We randomly chose M classifiers among the 21 (WNV) or 20 (SMR) classifiers used in the respective datasets to compute FiDEL scores $L_{\text{FiDEL}}$. Fig. 4 *A* and *B* shows the results of running FiDEL for the WNV data whereas Fig. 4 *C* and *D* shows the results for the SMR data. Fig. 4 *A* and *C* shows results for 200 randomly chosen sets of M=3, 5, and 7 classifiers. For each point in one such combination of classifiers, the *x* coordinate is the AUC of the best classifier and the *y* coordinate is the FiDEL AUC for that combination. In the vast majority of cases the points are above the identity line (dashed line), indicating that the FiDEL method outperforms the best among the M base classifiers in the vast majority of classifier choices for the ensemble, even when M=3 and more so for M=7. Fig. 4 *B* and *D* shows the average, over 200 combinations of M=3,…,10 randomly chosen classifiers, of the AUCs of the best individual classifier in the ensemble (gray dashed line), the WoC ensemble (black dashed line), and the FiDEL method (solid blue line). Error bars represent the SEM over the 200 combinations. FiDEL clearly and robustly outperforms both the WoC ensemble and the best individual classifier of the ensemble.

## Other Uses of the Class-Conditioned Rank Probability

In the previous sections, we argued that the FD distribution provides an explicit expression for the rank-conditioned class probability and its counterpart, the class-conditioned rank probability. In this section, we provide a few simple results that follow from expressing some performance metrics directly in terms of the class-conditioned rank probability. For example, in a previous section we demonstrated that the threshold for segregating the positive and the negative classes that zeroes the log-likelihood ratio is also the threshold that maximizes the balance accuracy, and we did so by expressing the balance accuracy in terms of the class-conditioned rank probabilities of the classifier. Using the same notation that we used earlier, let us denote by $y_{r}$ the true class of an item that a classifier placed at rank r. Given that $y_{r}$ can take only the values 1 and 0, its mean value $\left\langle y_{r}\right\rangle$ in the $\left(N,N_{1}\right)$ ensemble is equal to P(y=1|r). The false positive rate (FPR), the true positive rate (TPR) (also known as recall), the precision (Prec), and the balanced accuracy (bac), at a given rank k used as the threshold between positive and negative predicted classes, can all be computed as follows: $\text{TPR}\left(k\right)=1/N_{1}\sum_{r=1}^{k}y_{r}$, $\text{FPR}\left(k\right)=1/N_{0}\sum_{r=1}^{k}\left(1-y_{r}\right)$, $\text{Prec}\left(k\right)=1/k\sum_{r=1}^{k}y_{r}$, and bac(k)=(TPR(k)+1−FPR(k))/2. Therefore, their averages in the $\left(N,N_{1}\right)$ ensemble are

$$\left\langle \text{TPR}\left(k\right)\right\rangle =\frac{1}{N_{1}}\overset{k}{\underset{r=1}{\sum }}P\left(1|r\right);\left\langle r|1\right\rangle =\overset{N}{\underset{r=1}{\sum }}rP\left(r|1\right)$$ [27]

$$\left\langle \text{FPR}\left(k\right)\right\rangle =\frac{1}{N_{0}}\overset{k}{\underset{r=1}{\sum }}P\left(0|r\right);\left\langle r|0\right\rangle =\overset{N}{\underset{r=1}{\sum }}rP\left(r|0\right)$$ [28]

$$\left\langle \text{Prec}\left(k\right)\right\rangle =\frac{1}{k}\overset{k}{\underset{r=1}{\sum }}P\left(1|r\right)$$ [29]

$$\left\langle \text{bac}\left(k\right)\right\rangle =\frac{1}{2}\left(\left\langle \text{TPR}\left(k\right)\right\rangle +1-\left\langle \text{FPR}\left(k\right)\right\rangle \right).$$ [30]

Using these expressions, there are a number of interesting relations that can be derived *(**SI Appendix*). To start with, we can derive an expression for the AUC:

$$\left\langle AUC\right\rangle =\frac{\left\langle r|0\right\rangle -\left\langle r|1\right\rangle }{N}+\frac{1}{2}.$$ [31]

This relation is not new, as it can be obtained from the AUC relation to the Wilcoxon–Mann–Whitney *U* statistics, but in *SI Appendix* we derive it using the rank-conditioned class probability. Eq. **31** is interesting as it clearly shows that the AUC depends only on class-conditioned average ranks and not on other subtleties of the distribution of ranks.

A second interesting expression is

$$\left\langle AUC\right\rangle =2\bar{\left\langle \text{bac}\right\rangle }-\frac{1}{2},$$ [32]

where the overbar is the average over all thresholds: $\bar{\left\langle \text{bac}\right\rangle }=\frac{1}{N}\sum_{k=1}^{N}\left\langle \text{bac}\left(k\right)\right\rangle$. Eq. **32** relates the AUC and the average balance accuracy over all thresholds. As the maximum ⟨AUC⟩=1, Eq. **32** implies that the maximum $\bar{\left\langle \text{bac}\right\rangle }=3/4$.

A final interesting relation pertains the area under the precision recall curve (AUPRC):

$$\left\langle AUPRC\right\rangle =\frac{\rho }{2}\left(1+\frac{\bar{\left\langle \text{Prec}\left(k\right)\right\rangle \left\langle \text{Prec}\left(k+1\right)\right\rangle }}{\rho^{2}}\right)$$ [33]

$$\approx \frac{\rho }{2}\left(1+\frac{\bar{{\left\langle \text{Prec}\right\rangle }^{2}}}{\rho^{2}}\right),$$ [34]

where the approximation in Eq. **34** holds for N≫1. It is interesting that the AUPRC is related to the average square of the precision over all thresholds.

## Conclusion

The problem of binary classification is a fundamental task in machine learning. It has spurred the development of a wealth of ingenious algorithms including k-nearest neighbors, support vector machines, random forests, and deep learning to name but a few. Each of these algorithms outputs scores whose value depends on the intricacies of the algorithm and can be properly interpreted only in the narrow context in which the algorithm was used. However, when we try to combine algorithms with other elements of evidence to decide the class of an item, it would be desirable that the output of the algorithm be the probability that the item belongs to each class. Most algorithms do not have a way to compute well-calibrated class-conditioned score densities. Some methods, however, explicitly model the posterior probability of their classification, for example using logistic regression or Platt scaling methods (43), performing a logistic transformation of chosen features in the former or of a classifier score in the latter, into an output probability. While such transformations make intuitive sense and work well for some applications, they are heuristic methodologies. Our approach is different from the abovementioned methods on two counts: On the one hand, our logistic transformation transforms the ranks (not features or scores) assigned by a classifier to items in a test set into a probability; on the other hand, the logistic transformation is not postulated as an ad hoc transformation but results from the maximum-entropy principle and as such is the least-biased distribution given the information at hand. In other words, ours is the most parsimonious calibrated class distribution, and, in the absence of additional information, should be preferred to other methods.

In this paper, we address the problem of endowing any binary classifier with a probabilistic output using statistical physics considerations. We map the problem of estimating the probability that a classifier places a positive-class item at a given rank to the problem of computing the occupation number of a fermion in a given quantum state in a fermionic physical system with a finite number of single-fermion quantum states. This mapping leads to the identification of the rank-conditioned class probability of a classifier as the FD distribution describing an ensemble of fermionic systems. The FD distribution depends on two parameters of the physical system: the temperature and the chemical potential. We showed that the interpretation of these parameters in a fermionic system can be useful in understanding the role of these same parameters in the classification problem: In physics the temperature is a manifestation of how disordered a system can be whereas in a classification problem the temperature measures how far a classifier is from the perfect classifier. A temperature of 0 implies a perfect classifier and a temperature of infinity results in a random classifier. The chemical potential measures the energy at which the occupation number of fermions is 50/50, and therefore in the classification system it is related to the rank threshold that separates predicted positive and negative classes. Having a precise functional form for the rank-conditioned class probability allowed us to calculate the optimal threshold to separate predicted positive and negative classes. It also permitted the calculation of the SD of the AUC necessary to estimate confidence intervals and perform power analyses. By way of estimating the class probabilities in rank space, our formalism provides a calibrated class probability that can be used to combine classifiers. This allowed us to propose the ensemble learning algorithm that we call FiDEL. We also showed that expressing performance metrics in terms of rank-conditioned class probabilities is a useful tool for formal derivations: for example, the derivation that the threshold that best separates predicted classes using the likelihood-ratio method is also the threshold that maximizes the balanced accuracy of a classification.

Many of the ideas presented in this paper are of a theoretical nature. However, we can envision practical applications of our theory that can be implemented relatively easily. As an example, suppose that we have dataset such as the one used in ref. 9, consisting of a collection of screening mammograms from women whose breast cancer status after the screening examination is known to be positive or negative. Assume that we divide this set into two partitions: a training set with, e.g., 50% of the data and a validation set with the remaining 50%. After training our classifier in the training set, we compute the AUC and the prevalence ρ in the validation set, from which we derive the FD parameters β and μ. When a woman goes to the radiologist for her next breast cancer screening examination, our classifier processes the mammogram yielding a score, from which we find its rank in the context of the other scores in the validation set. In this way we find the rank order r of the new mammogram in the validation set. We then use the FD distribution with the parameters obtained from the validation set to compute the probability that this woman has cancer according to the classifier. This calibrated probability that a woman has cancer given her mammogram and the score outputted by the given classifier in the context of a validation set can be used by radiologists as a decision aid to decide whether a woman must be recalled or not for further studies after screening. Given that the FD distribution is the maximum-entropy distribution, this probability is the most unbiased estimate given the data at hand. Similar strategies can be envisioned in other application domains where a validation set and a preferred classifier are available.

It is important to highlight limitations of our approach. To start with, we need to be clear that the FD distribution is not, in general, the exact rank-conditioned class probability of a classifier in a test set. It is, however, the probability distribution that is maximally noncommittal about the aspects of the problem we have no information on, but does include the information that we have about our problem, namely the classifier AUC, the fraction of positive examples, and the total number of elements in the test set. If we had more information about the distribution of scores, or if we had the area under the precision-recall curve, for example, then we could improve the rank-conditioned class probability beyond the FD distribution. A second consideration is that the FD distribution represents the probability that items at a given rank are in the positive class in an ensemble of specific characteristics (the $\left(N,N_{1}\right)$ ensemble of test sets). However, in typical applications we have just one test set and we find the FD parameters from one instance of the ensemble. This means that we have a single AUC estimate from one test set and use that point estimate as an estimator for the average AUC. Furthermore, in typical applications, we do not know the labels in the test set, and therefore we cannot compute the AUC in the test set. In these cases, we need to use the AUC as well as the fraction of positive examples ρ from a validation set as we did in the WNV and SMR classification problems presented in this paper. Finally, the ensemble learning algorithm we proposed was derived under the assumption that the base classifiers are class-conditionally independent, which is a strong assumption that holds only approximately. However, we showed that the FiDEL ensemble overperforms the best of the base classifiers even if there is moderate class-conditioned correlation among the base classifiers up to an average correlation of 0.4 to 0.5. We also showed that training base classifiers in disjoint partitions of a dataset, or in completely different datasets, such as in federated learning, would reduce the dependence between classifiers. We are exploring possible modifications to FiDEL that take into account the correlation between base classifiers in an ensemble.

Despite some of these limitations, we believe that the FD distribution is a useful tool to model the rank-conditioned class probability of a classifier. By transforming scores into ranks, the FD distribution provides a calibrated probabilistic output for binary classifiers.

## Materials and Methods

### Datasets.

We used datasets for binary classification problems from two Kaggle competitions: The WNV Prediction challenge and the SMR challenge. The WNV competition, which took place in 2015, challenged participants to predict the presence or absence of West Nile virus across the city of Chicago based on tests performed on mosquitoes caught in traps. The data provided to make those predictions included record identification (id), date, address, mosquito species, trap id, and number of mosquitoes. Participants were also given weather data concurrent with the mosquito testing period (2007 to 2014) and the date and location of chemical spraying conducted by the city during 2011 and 2013. The SMR competition ran in 2015 and challenged participants to predict whether or not customers will respond to a marketing mail offer sent to them. Each row corresponds to one customer with 1,934 anonymized features composed of a mix of continuous and categorical variables. More detail can be found in *SI Appendix*, Table S1 and section 9.

### Classifiers.

The classifiers used in each of the competitions are described in *SI Appendix*, Table S2. A total of 23 classifiers were used of which 21 classifiers were used in the WNV dataset and 20 classifiers in the SMR dataset.

### Statistical Analysis and Visualization.

Statistical analysis and visualization were performed using R (http://www.R-project.org). Source code can be found at https://github.com/sungcheolkim78/FiDEL.

## Supplementary Material

Supplementary File

## Data Availability

All study data are included in this article, and/or *SI Appendix*, and/or in GitHub, https://github.com/sungcheolkim78/FiDEL/tree/master/kaggle/data (41, 42).
