# Supplementary material for: The Fermi–Dirac distribution provides a calibrated probabilistic output for binary classifiers
Source: Proc Natl Acad Sci U S A. 2021 Aug 19;118(34):e2100761118. doi: 10.1073/pnas.2100761118 (PMC8403970; doi:10.1073/pnas.2100761118)
Supplement: Supplementary File [file pnas.2100761118.sapp.pdf]

PNAS
www.pnas.org

Supplementary Information for

The Fermi Dirac Statistics Provides a Calibrated Probabilistic Output for Binary Classifiers

Sung-Cheol Kim, Adith S. Arun, Mehmet Eren Ahsen, Robert Vogel and Gustavo Stolovitzky

Gustavo Stolovitzky
E-mail: gustavo@us.ibm.com

This PDF file includes:

Supplementary text
Figs. S1 to S4
Tables S1 to S2
SI References

Supporting Information Text

1. Problem Setup

A. The (N, N_1)-ensemble of test sets. Given a classification problem, we assume that each classifiable item can be characterized with an instance pair $(X, Y) \in \mathcal{X} \times \{0, 1\}$. This pair is a random vector with a well-defined probability density function. The set $\mathcal{X}$ denotes the feature space, and we will assume w.l.o.g. that $y = 0$ denotes the negative class and $y = 1$ denotes the positive class. Let $\Omega(N, N_1) = \{(x_1, y_1), \cdots, (x_N, y_N) \in (\mathcal{X} \times \{0, 1\})^N : \sum_{i=1}^{N} y_i = N_1\}$ denote the space of all N i.i.d realizations of $\mathcal{X} \times \{0, 1\}$ with exactly N_1 positive instances. We will call this set the (N, N_1)-ensemble of test sets. We call these test sets because when we are given a realization $T \in \Omega(N, N_1)$, we assume that we do not observe the labels associated with each sample and only observe the feature vector x_k associated with each sample. All instances $T \in \Omega(N, N_1)$ have the same fraction of positive examples, which we will call the prevalence (of the positive class) and denote by ρ.

B. Classification of a test set in the (N, N_1)-ensemble results in N ranked items. Let g represent a classifier that maps the feature space $\mathcal{X}$ to the real numbers. We assume that classifier g is already trained and produces N scores $\{g(x_k)\}_{k=1}^{N}$ when applied to a realization $T = \{(x_k, y_k)\}_{k=1}^{N}$ of the (N, N_1)-ensemble, with unknown class labels $\{y_k\}_{k=1}^{N}$. We will use the rank transformation as a calibration tool due to its theoretical implications presented in the paper. For each instance $T \in \Omega(N, N_1)$ and classifier g, consider the following vector $g(T) = [g(x_1), \cdots, g(x_N)]^T \in \mathbb{R}^N$. By using the well-ordering property of the real numbers, we can map $g(T)$ to a rank vector $\mathbf{r} = [r_1, ..., r_N]^T \in \mathcal{S}_N$, where $\mathcal{S}_N$ denotes the set of all permutations of the integers $\{1, \cdots, N\}$. We assume that ties, i.e. $g(x_j) = g(x_k)$ for $j \neq k$, are broken uniformly at random.

2. Class-conditioned rank probability and rank-conditioned class probability

A. Notation. With the above problem setup, we let $\text{Prob}(R = r|Y = y)$ denote the probability, over all realizations from $\Omega(N, N_1)$, that a sample of class $y \in \{0, 1\}$ has been assigned rank $r \in \{1, \cdots, N\}$ by classifier g. Analogously, let $\text{Prob}(Y = y|R = r)$ denote the probability, over all realizations from $\Omega(N, N_1)$, that the true label of an item placed at rank r by classifier g is y. We will use the following shorthand notation:

$$P(r|y) = \text{Prob}(R = r|Y = y);\ P(r|1) = \text{Prob}(R = r|Y = 1);\ P(r|0) = \text{Prob}(R = r|Y = 0);$$
$$P(y|r) = \text{Prob}(Y = y|R = r);\ P(1|r) = \text{Prob}(Y = 1|R = r);\ P(0|r) = \text{Prob}(Y = 0|R = r).$$

We will call $P(r|y)$ the class-conditioned rank probability and $P(y|r)$ the rank-conditioned class probability.

The conditional expected value of R given the class in the (N, N_1)-ensemble will be denoted by $\langle r|y \rangle$, and is computed from the corresponding probabilities:

$$\langle r|1 \rangle = \sum_{r=1}^{N} r P(r|1),$$

$$\langle r|0 \rangle = \sum_{r=1}^{N} r P(r|0).$$

B. Calculation of the rank-conditioned class and class-conditioned rank probabilities. Theoretically, at least, it is possible to design a direct way to calculate $P(y|r)$ for a classifier. To do that, we create a finite (N, N_1)-ensemble with $L \gg 1$ instances $T_{i=1}^{L}$. We apply the classifier to each of the instances $T_{i=1}^{L}$ and for instance T_i we create a ranked list $r_{ik} \in \{1, 2, ..., N\}$ as discussed in 1B. The rank-conditioned positive-class probability $P(1|r)$ in this case is

$$P(1|r) = \frac{1}{L} \sum_{i=1}^{L} y_{ik(r)}, \qquad [1]$$

where $y_{ik(r)}$ is the label of the item $k(r)$ that was placed at rank r in test set instance T_i. In other words, $P(1|r)$ is the frequency with which classifier g places positive class items at rank r. Eq. (1) is conceptual, as in most cases we won't have the labels y_{ik} of the test sets. However, when we have the labels, as in the simulations done in Fig. 1, $P(1|r)$ is computed as prescribed using Eq. (1).

We can calculate the class-conditioned rank probability $P(r|y)$ from $P(y|r)$ using Bayes' theorem:

$$P(r|y) \cdot P(Y = y) = P(y|r) \cdot P(R = r),$$

where $P(R = r) = \frac{1}{N}$, $P(Y = 1) = \rho$ and $P(Y = 0) = 1 - \rho$. Therefore,

$$P(r|1) = P(1|r)/N_1, \text{ and } P(r|0) = P(0|r)/(N - N_1).$$

3. Performance metrics and the rank-conditioned class probability

We showed in the main text that the average value of the traditional binary classification performance metrics like the true positive rate (TPR), false positive rate (FPR), precision (Prec) and balanced accuracy (bac) can be expressed in terms of the rank-conditioned class probability:

$$\langle \mathrm{TPR}(k)\rangle = \frac{1}{N_1}\sum_{r=1}^{k} P(1|r), \tag{2}$$

$$\langle \mathrm{FPR}(k)\rangle = \frac{1}{N_0}\sum_{r=1}^{k} P(0|r), \tag{3}$$

$$\langle \mathrm{Prec}(k)\rangle = \frac{1}{k}\sum_{r=1}^{k} P(1|r), \tag{4}$$

$$\langle \mathrm{bac}(k)\rangle = \frac{1}{2}\left(\langle \mathrm{TPR}(k)\rangle + 1 - \langle \mathrm{FPR}(k)\rangle\right). \tag{5}$$

In this section, we will show that the area under the ROC curve (AUC) can be expressed in terms of the class-conditioned averages of the ranks. These relationships are known in statistics in terms of the Wilcoxon-Mann-Whitney U test for a sample. Here we will re-derive them using rank-conditioned class probabilities as averages in the (N, N_1)-ensemble. We will also show that the $\langle AUC\rangle$ of a classifier can be expressed in terms of an average over all ranks of the average balanced accuracy in the (N, N_1)-ensemble. Finally, we will characterize the average area under the precision-recall curve $\langle AUPRC\rangle$ in terms of an average over all possible thresholds using the second moment of the precision.

A. Expressing the $\langle AUC\rangle$ in terms of class-conditioned averages. To compute the $\langle AUC\rangle$, we will use the trapezoidal rule to integrate the ROC curve. Using k as a parameter, the ROC curve can be expressed as the set of points $(\langle \mathrm{FPR}(k)\rangle, \langle \mathrm{TPR}(k)\rangle)$ with $k = 0, \cdots, N$ and $\langle \mathrm{TPR}(0)\rangle = \langle \mathrm{FPR}(0)\rangle = 0$:

$$\langle AUC\rangle = \sum_{k=0}^{N-1} \frac{\langle \mathrm{TPR}(k)\rangle + \langle \mathrm{TPR}(k+1)\rangle}{2} \cdot \triangle\langle \mathrm{FPR}(k)\rangle, \tag{6}$$

where we used the the forward difference operator $\triangle$ defined as $\triangle f(k) = f(k+1) - f(k)$. Before stating the main result of this subsection (Theorem 1), it will be useful to prove a few auxiliary results.

Lemma 1. The sum of the $\langle \mathrm{TPR}(k)\rangle$ and $\langle \mathrm{FPR}(k)\rangle$ over all thresholds k is related to the class-conditioned expected rank:

$$\sum_{k=1}^{N} \langle \mathrm{TPR}(k)\rangle = N + 1 - \langle r|1\rangle, \tag{7}$$

$$\sum_{k=1}^{N} \langle \mathrm{FPR}(k)\rangle = N + 1 - \langle r|0\rangle. \tag{8}$$

Proof. Rewriting the sum in Eq. (7) using Eq. (2), and recalling that $\sum_{k=1}^{N}\sum_{i=1}^{k} f(i) = \sum_{i=1}^{N}\sum_{r=k}^{N} f(r)$, results in

$$\sum_{k=1}^{N} \langle \mathrm{TPR}(k)\rangle = \sum_{k=1}^{N} \frac{1}{N_1}\sum_{r=1}^{k} P(1|r) = \frac{1}{N_1}\sum_{r=1}^{N}\sum_{k=r}^{N} P(1|r) \tag{9}$$

$$= \frac{1}{N_1}\sum_{r=1}^{N} (N + 1 - r) \cdot P(1|r) \tag{10}$$

$$= N + 1 - \langle r|1\rangle \tag{11}$$

which proves Eq. (7). We can prove Eq. (8) following a similar derivation. However, let's prove it by way of a symmetry argument. Let us swap the names of the classes such that what we called the positive class before is now our negative class, and vice versa. The items $1 \leq r \leq k$ that were positives are now negatives, and therefore the $\mathrm{TPR}(k)$ becomes the $\mathrm{FPR}(k)$. Therefore, by changing TPR for FPR and 1 for 0 in Eq. (7) we get Eq. (8). □

We can now state the following result.

Theorem 1. *The average $\langle AUC\rangle$ of a classifier in the (N, N_1)-ensemble can be expressed in terms of the class-conditioned expectation of the ranks as*

$$\langle AUC\rangle = \frac{\langle r|0\rangle - \langle r|1\rangle}{N} + \frac{1}{2}, \tag{12}$$

and the average sum of the rank of the positive class, $N_1\langle r|1\rangle$*, can be expressed in terms of the* $\langle AUC\rangle$ *as*

$$N_1\langle r|1\rangle = \frac{N+1}{2}N_1 - N_0N_1\left(\langle AUC\rangle - \frac{1}{2}\right). \qquad [13]$$

Proof. This derivation extends to averages in the (N, N_1)-ensemble, a similar result proved in (1) for one realization of the test set. We rewrite Eq. (6), using from Eq. (2) and Eq. (3) that $\triangle\langle \text{TPR}(k)\rangle = P(1|k+1)/N_1$ and $\triangle\langle \text{FPR}(k)\rangle = P(0|k+1)/N_0 = (1 - P(1|k+1))/N_0$, as follows

$$\begin{aligned}\langle AUC\rangle &= \frac{1}{2N_0}\sum_{k=0}^{N-1}(\langle \text{TPR}(k)\rangle + \langle \text{TPR}(k+1)\rangle)\cdot(1 - P(1|k+1)) \\ &= \frac{1}{2N_0}\sum_{k=0}^{N-1}(\langle \text{TPR}(k)\rangle + \langle \text{TPR}(k+1)\rangle)\cdot(1 - N_1\triangle\langle \text{TPR}(k)\rangle),\end{aligned}$$

After expanding the product inside the sum, the last equation can be parsed into two halves. Operating over the first half we get

$$\begin{aligned}\frac{1}{2N_0}\sum_{k=0}^{N-1}(\langle \text{TPR}(k)\rangle + \langle \text{TPR}(k+1)\rangle) &= \frac{1}{2N_0}\left[\left(2\sum_{k=0}^{N}\langle \text{TPR}(k)\rangle\right) - \langle \text{TPR}(0)\rangle - \langle \text{TPR}(N)\rangle\right] \\ &= \frac{1}{2N_0}\left(2\sum_{k=0}^{N}\langle \text{TPR}(k)\rangle - 1\right),\end{aligned}$$

where we used that $\langle \text{TPR}(0)\rangle = 0$ and $\langle \text{TPR}(N)\rangle = 1$. Operating over the second half we get:

$$\begin{aligned}\frac{1}{2N_0}\sum_{k=0}^{N-1}(\text{TPR}(k) + \text{TPR}(k+1))\cdot\triangle\text{TPR}(k) &= \frac{1}{2N_0}\sum_{k=0}^{N-1}(\text{TPR}(k+1)^2 - \text{TPR}(k)^2) \\ &= \frac{N_1}{2N_0},\end{aligned}$$

where we used that the sum over the difference of squares in the first line of the last equation is a telescopic sum, and that $\langle \text{TPR}(0)\rangle = 0$ and $\langle \text{TPR}(N)\rangle = 1$. Adding the two halves results in the following expression for the $\langle AUC\rangle$:

$$\langle AUC\rangle = \frac{1}{2N_0}\left(2\sum_{k=1}^{N}\langle \text{TPR}(k)\rangle - 1\right) - \frac{N_1}{2N_0}.$$

Using Lemma 1 we obtain

$$\begin{aligned}\langle AUC\rangle &= \frac{1}{2N_0}\left(2N + 1 - 2\langle r|1\rangle\right) - \frac{N_1}{2N_0} \\ &= \frac{2N+1-N_1}{2N_0} - \frac{\langle r|1\rangle}{N_0}. \qquad [14]\end{aligned}$$

Similarly, we can re-write $\langle AUC\rangle$ in terms of $\langle r|0\rangle$ by a symmetry argument. If the items that were called positives are now called negatives (and vice versa) then 1 changes to 0, 0 changes to 1, the $\langle AUC\rangle$ changes to $1 - \langle AUC\rangle$, N_1 changes to N_0 and N_0 changes to N_1. Doing those changes in Eq. (14) and after some algebra we get

$$\langle AUC\rangle = \frac{\langle r|0\rangle}{N_1} - \frac{N_0+1}{2N_1}. \qquad [15]$$

Multiplying Eq. (14) times N_0 and Eq. (15) times N_1 and adding the resulting equations we get:

$$(N_1 + N_0)\langle AUC\rangle = \langle r|0\rangle - \frac{N_0+1}{2} + \frac{2N+1-N_1}{2} - \langle r|1\rangle$$

from where

$$\langle AUC\rangle = \frac{\langle r|0\rangle - \langle r|1\rangle}{N} + \frac{1}{2}$$

which proves Eq. (12). Eq. (13) follows from Eq. (14) after multiplying it by N_1 and rearranging the terms. □

B. Expressing the $\langle AUC\rangle$ and $\langle AUPRC\rangle$ in terms of averages over all thresholds. The balanced accuracy of a classifier at threshold rank k can be written as

$$\langle \mathrm{bac}(k)\rangle \quad = \quad \frac{1}{2}(\langle \mathrm{TPR}(k)\rangle + 1 - \langle \mathrm{FPR}(k)\rangle).$$

Using the rank-conditioned class probability, we can express the $\langle AUC\rangle$ of a classifier in terms of the average balance accuracy over all possible thresholds.

Theorem 2. *The average $\langle AUC\rangle$ in the (N, N_1)-ensemble can be expressed in terms of the average balanced accuracy of a classifier according to*

$$\langle AUC\rangle = 2\overline{\langle \mathrm{bac}\rangle} - \frac{1}{2}$$

where $\overline{\langle \mathrm{bac}\rangle} = \sum_{k=1}^{N}\langle \mathrm{bac}(k)\rangle/N$

Proof. From lemma 1,

$$\langle r|1\rangle = N + 1 - \sum_{k=1}^{N}\langle \mathrm{TPR}(k)\rangle \quad \text{and} \quad \langle r|0\rangle = N + 1 - \sum_{k=1}^{N}\langle \mathrm{FPR}(k)\rangle.$$

Subtracting $\langle r|1\rangle$ from $\langle r|0\rangle$ from the previous equation we get

$$\langle r|0\rangle - \langle r|1\rangle = \sum_{k=1}^{N}\langle \mathrm{TPR}(k)\rangle - \sum_{k=1}^{N}\langle \mathrm{FPR}(k)\rangle = \sum_{k=1}^{N}(2\langle \mathrm{bac}(k)\rangle - 1).$$

Using theorem 1 we can express $\langle r|0\rangle - \langle r|1\rangle$ in terms of $\langle AUC\rangle$ in the previous equation, from where

$$\langle AUC\rangle = 2\left(\frac{1}{N}\sum_{k=1}^{N}\langle \mathrm{bac}(k)\rangle\right) - \frac{1}{2},$$

which proves the theorem. □

Next, we use the rank-conditioned class probability to characterize the average area under the precision-recall curve (AUPRC) in terms of the average squared precision over all thresholds. The precision at threshold rank k is defined in Eq. (4). We will integrate the precision vs recall curve (PRC) using the trapezoidal rule:

$$\langle AUPRC\rangle = \sum_{k=0}^{N-1}\frac{\langle \mathrm{Prec}(k)\rangle + \langle \mathrm{Prec}(k+1)\rangle}{2}\cdot \triangle\langle \mathrm{TPR}(k)\rangle, \tag{16}$$

where by definition $\langle \mathrm{Prec}(0)\rangle = 1$. We can now prove the following:

Theorem 3. *The average $\langle AUPRC\rangle$ over the (N, N_1)-ensemble can be written as*

$$\langle AUPRC\rangle = \frac{\rho}{2} + \frac{\overline{\langle \mathrm{Prec}(k)\rangle\langle \mathrm{Prec}(k+1)\rangle}}{2\rho}, \tag{17}$$

where $\overline{\langle \mathrm{Prec}(k)\rangle\langle \mathrm{Prec}(k+1)\rangle} = \sum_{k=0}^{N-1}\langle \mathrm{Prec}(k)\rangle\langle \mathrm{Prec}(k+1)\rangle/N$. *When $N \gg 1$, the $\langle AUPRC\rangle$ can be approximated by*

$$\langle AUPRC\rangle \approx \frac{\rho}{2} + \frac{\overline{\langle \mathrm{Prec}\rangle^2}}{2\rho}, \tag{18}$$

where $\overline{\langle \mathrm{Prec}\rangle^2} = \sum_{k=1}^{N}\langle \mathrm{Prec}(k)\rangle^2/N$.

Proof. Let's define the function $f(k)$ as

$$f(k) = k\ \langle \mathrm{Prec}(k)\rangle = \sum_{r=0}^{k} P(1|r).$$

Using the forward difference operator $\triangle f(k) = f(k+1) - f(k)$ we get

$$\triangle f(k) = (k+1)\langle \mathrm{Prec}(k+1)\rangle - k\langle \mathrm{Prec}(k)\rangle = P(1|k+1)$$

From Eq. (16), the $\langle AUPRC \rangle$ can be written as

$$\begin{aligned}
\text{AUPRC} &= \sum_{k=0}^{N-1} \frac{1}{2}\left(\frac{f(k+1)}{k+1} + \frac{f(k)}{k}\right) \cdot \frac{\triangle f(k)}{N_1} \\
&= \frac{1}{2N_1}\sum_{k=0}^{N-1}\left(\frac{f(k+1)}{k+1} + \frac{f(k)}{k}\right)(f(k+1) - f(k)) \\
&= \frac{1}{2N_1}\sum_{k=0}^{N-1}\left(\frac{f(k+1)^2}{k+1} - \frac{f(k)^2}{k} + \frac{f(k)f(k+1)}{k(k+1)}\right) \\
&= \frac{1}{2N_1}\left(N\,\langle \text{Prec}(N)\rangle^2 - 0 \cdot \langle \text{Prec}(0)\rangle^2\right) + \frac{1}{2N_1}\sum_{k=0}^{N-1}\langle \text{Prec}(k)\rangle\langle \text{Prec}(k+1)\rangle \\
&= \frac{\rho}{2} + \frac{1}{2\rho}\overline{\langle \text{Prec}(k)\rangle\langle \text{Prec}(k+1)\rangle},
\end{aligned}$$

where we used the notation $\overline{g} = \frac{1}{N}\sum_{k=0}^{N-1} g(k)$. This proves the first part of the theorem.

When $N \gg 1$, we can approximate the finite sums for integrals, to get

$$\langle \text{Prec}(k)\rangle \approx \frac{1}{k}\int_1^k P(1|r)dr, \quad [19]$$

$$\langle \text{TPR}(k)\rangle \approx \frac{1}{N_1}\int_1^k P(1|r)dr, \quad [20]$$

and

$$\langle AUPRC \rangle \approx \int_1^N \langle \text{Prec}(r)\rangle d\langle \text{TPR}(r)\rangle$$

We now insert Eq. (19) and Eq. (20) in the previous equation, and integrate by parts:

$$\begin{aligned}
\langle AUPRC \rangle &\approx \int_1^N \frac{1}{r}\left(\int_1^r P(1|x)dx\right)\frac{1}{N_1}P(1|r)dr \\
&= \frac{1}{2N_1}\int_1^N \frac{1}{r}\frac{d}{dr}\left(\int_1^r P(1|x)dx\right)^2 dr \\
&= \frac{1}{2N_1}\left[\frac{1}{r}\left(\int_1^r P(1|x)dx\right)^2\right]_1^N + \frac{1}{2N_1}\int_1^N \left(\frac{1}{r}\int_1^r P(1|x)dx\right)^2 dr \\
&= \frac{\rho}{2} + \frac{\overline{\langle \text{Prec}\rangle^2}}{2\rho},
\end{aligned}$$

where for the last equation we used $\overline{g} = \frac{1}{N}\int_1^N g(x)dx \approx \frac{1}{N}\sum_{k=1}^N g(k)$. This proves the second part of the theorem. □

4. Fermi-Dirac Distribution as the maximum-entropy estimate of the class probability given the $\langle AUC \rangle$ and ρ

We made the point in the main text that the mapping of fermionic systems to binary classification and the fact that the FD distribution is the maximum entropy distribution in the physical sense for the fermionic system justified the choice of the FD distribution as the least biased distribution of the rank-conditioned class probability given the constraints. That the maximum entropy from a physics point of view corresponds to a distribution that has the maximum uncertainty given the information at hand has been argued in the seminal 1957 paper by Jaynes (2). Jaynes argued that statistical mechanics can be viewed as a form of statistical inference rather than as a physical theory, and all of statistical mechanics can be derived from maximizing the Shannon Entropy. Therefore, it should be possible for us to derive that the rank-conditioned rank distribution introduced here coincides with the FD distribution when we maximize Shannon entropy with the appropriate constraints. This will justify using the FD distribution in binary classification, not as the true rank-conditioned class probability, but as the distribution that is maximally non-committal with regard to missing information. In this section we derive such a distribution. Except for minor details in the treatment of the constraints, this derivation is similar to the derivation of the rank-conditioned class probability we advanced in the context of the SUMMA algorithm for unsupervised ensemble learning in (1).

Our starting point is to define the probability of having items in a class vector $(y_1, y_2, \ldots, y_N)$ with a class y_r at rank $r \in (1, 2, \ldots, N)$. For example, a given realization of a test set from the (N, N_1)-ensemble may have an item of class 1 at rank 1 ($y_1 = 1$), an item of class 0 at rank 2 ($y_2 = 0$), an item of class 1 at rank 3 ($y_3 = 1$), etc., up to an item of class 0 at rank N ($y_N = 0$). For this realization, the class vector will be $(1, 0, 1, \ldots, 0)$. We will call $Q(y_1, y_2, \ldots, y_N)$ the probability of class vector $(y_1, y_2, \ldots, y_N)$.

We will assume that the probability that there is an item of class y_r at rank r is independent of the probability that there is an item of rank y_k at rank k. Therefore the probability of the class vector factorizes

$$Q(y_1, y_2, \dots, y_N) = P(y_1|1)\dots P(y_N|N),$$

where $P(y_r|r)$ is the rank-conditioned class probability at rank r.

The entropy of the probability of the class vector is

$$S(Q) = -\sum Q \ln Q$$

and using its factorization, we obtain

$$\begin{aligned} S(Q) &= -\sum_{y_1=0,1}\sum_{y_2=0,1}\dots\sum_{y_N=0,1} P(y_1|1)\dots P(y_N|N)(\ln P(y_1|1) + \dots + \ln P(y_N|N)) \\ &= -\left(\sum_{y_1=0,1} P(y_1|1)\ln P(y_1|1) + \dots + \sum_{y_N=0,1} P(y_N|N)\ln P(y_N|N)\right) \\ &= \sum_{r=1}^{N}\sum_{y_r=0,1} P(y_r|r)\ln P(y_r|r). \end{aligned}$$

If we did not know anything else about the problem (except for the obvious fact that each $P(0|r)+P(1|r)=1$), maximizing the above entropy would yield $P(0|r) = P(1|r) = 1/2$, in agreement with Laplace's intuition in his "Principle of Insufficient Reason", which stated that two events are to be assigned equal probabilities if there is no reason to think otherwise. But in our case there is reason to think otherwise. Besides the normalization constraint $P(0|r)+P(1|r)=1$ for each r, we know the average number of class 1 items in the (N, N_1)-ensemble is N_1. That is a property of the prevalence ρ of class 1 items in the dataset, as $N_1 = N\rho$. There is another constraint we can include, which is a property of the classifier's performance, which is related to the average rank of class 1 items. These two conditions (the one expressing a property of the dataset and the other expressing a property of the classifier) and the normalization conditions are our constraints for $P(y|r)$. These constraints can be written as follows:

$$\sum_{y=0,1} P(y|1) = 1; \sum_{y=0,1} P(y|2) = 1; \dots \sum_{y=0,1} P(y|N-1) = 1; \sum_{y=0,1} P(y|N) = 1; \tag{21}$$

$$N_1 = N\rho = \sum_{r=1}^{N}\sum_{y_r=0,1} y_r P(y_r|r) \tag{22}$$

$$R = \langle \sum_{r:y_r=1} r \rangle = N_1\langle r|1\rangle = \sum_{r=1}^{N}\sum_{y_r=0,1} y_r r P(y_r|r). \tag{23}$$

R is the average sum of the ranks r on the (N, N_1)-ensemble for which $y_r = 1$ and is equal to $N_1\langle r|1\rangle$. We already showed (Eq. (13) in Theorem 1) that R can be written in terms of $\langle AUC\rangle$. These constraints need to be added with their corresponding Lagrange multipliers in the expression to be maximized

$$\mathcal{C}(Q) = S(Q) + \sum_{r=1}^{N}\alpha_r(1 - \sum_{y_r=0,1} P(y_r|r)) \tag{24}$$

$$+\lambda(N_1 - \sum_{r=1}^{N}\sum_{y_r=0,1} y_r P(y_r|r)) + \beta(N_1\langle r|1\rangle - \sum_{r=1}^{N}\sum_{y_r=0,1} r y_r P(y_r|r)). \tag{25}$$

To find the set of $P(y|r)$'s that maximizes the entropy with the given constraints, we take the variations of $\mathcal{C}(Q)$ by perturbing each $P(y|r)$ with arbitrary variation $\delta P_r := \delta P(y|r)$ with respect to each $P(y|r)$

$$\delta\mathcal{C}(Q) = -\sum_{y=0,1}\delta P_r\left[\ln P(y|r) + 1 + \alpha_r + \lambda y + \beta y r\right] = 0.$$

As the variations δP_r are arbitrary, the terms in brackets have to be 0, that is

$$P(1|r) = \exp\left(-\alpha_r - 1 - \lambda - \beta r\right), \quad \text{and} \quad P(0|r) = \exp\left(-\alpha_r - 1\right).$$

The constants α_r are determined from the constraints in Eq. (21), from where

$$P(1|r) = \frac{1}{1 + e^{\beta(r-\mu)}}$$

where $\mu = \lambda/\beta$. The constants β and μ are determined using Eq. (22) and Eq. (23) respectively. It needs to be re-stated that we do not claim that the FD distribution is the true rank-conditioned class distribution. However, as is the case with distributions resulting from a maximum entropy argument, it is the most non-committal, and therefore least biased distribution that we can use when only the given constraints are known.

5. Calculation of β and μ

As discussed in the main text, the parameters β and μ are calculated from the two constraints on the rank-conditioned class probability:

$$\sum_{r=1}^{N} \frac{1}{1+e^{\beta(r-\mu)}} = N_1 \qquad [26]$$

$$\sum_{r=1}^{N} \frac{r}{1+e^{\beta(r-\mu)}} = N_1 \frac{N+1}{2} + N_1(N-N_1)(\frac{1}{2} - \langle AUC \rangle). \qquad [27]$$

We now re-scale r with N to generate the new variable $\xi = r/N$ and call $\beta' = \beta N$ and $\mu' = \mu/N$. Dividing the above equations by N, calling $d\xi = 1/N$, and approximating the sums by integrals (which is justified for sufficiently large $N \gg 1$), we can rewrite Eqs. (48) and (49) as

$$\int_0^1 \frac{d\xi}{1+e^{\beta'(\xi-\mu')}} \approx \rho, \qquad [28]$$

$$\int_0^1 \frac{\xi \; d\xi}{1+e^{\beta'(\xi-\mu')}} \approx \frac{\rho}{2} + \rho(1-\rho)(\frac{1}{2} - \langle AUC \rangle). \qquad [29]$$

Integrating Eq. (28) we get

$$\begin{aligned} \rho &= \left.\frac{\beta'\xi - \log\left(e^{\beta'(\xi-\mu')}+1\right)}{\beta'}\right|_0^1 \\ &= 1 - \frac{\log\left(e^{\beta'(1-\mu')}+1\right)}{\beta'} + \frac{\log\left(e^{-\beta'\mu'}+1\right)}{\beta'} \\ &=1 - \frac{\log\left(e^{\beta'(1-\mu')}+1\right)}{\beta'} + \frac{\log\left(e^{-\beta'\mu'}+1\right)}{\beta'}, \end{aligned}$$

from where μ' can be expressed as a function of ρ and β':

$$\mu' = \frac{1}{2} - \frac{1}{\beta'} \ln\left(\frac{\sinh \beta'(1-\rho)/2}{\sinh \beta'\rho/2}\right). \qquad [30]$$

In like manner, Eq. (29) can be integrated to yield

$$\frac{\rho}{2} + \rho(1-\rho)(\frac{1}{2} - \langle AUC \rangle) = \left.\frac{\mathrm{Li}_2\left(-e^{-\beta(\xi-\mu)}\right) - \beta\xi\log\left(e^{-\beta(\xi-\mu)}+1\right)}{\beta^2}\right|_0^1 \qquad [31]$$

$$= \frac{\mathrm{Li}_2\left(-e^{-\beta(1-\mu)}\right) - \mathrm{Li}_2\left(-e^{\beta\mu}\right) - \beta\log\left(e^{-\beta(1-\mu)}+1\right)}{\beta^2}, \qquad [32]$$

where $\mathrm{Li}_2(z)$ is the dilogarithm function. A general analytical expression to express β' and μ' in terms of ρ and $\langle AUC \rangle$ does not exist. Therefore given ρ and $\langle AUC \rangle$ we numerically solve for β' using Eq. (32) after expressing μ' in terms of β' and ρ using Eq. (30). After solving for β', we use Eq. (30) to find μ'. The sought for parameters β and μ can then be obtained from β' and μ' from the knowledge of N:

$$\beta = \beta'/N; \quad \text{and} \quad \mu = \mu' N.$$

In some some limiting cases, such as $\beta' \ll 1$, $\beta' \gg 1$ and $\rho = 1/2$ (see next subsections) it is possible to express β' and μ' explicitly in terms of ρ and $\langle AUC \rangle$.

A. Case $\beta \ll 1$. The case $\beta \ll 1$ corresponds to weak classifiers (high temperature) for which the rank-conditioned class probability has a very weak dependence on the rank r or in the re-scaled version, on ξ. In this case we can expand $\frac{1}{1+e^{\beta'(\xi-\mu')}}$ around $\xi = 1/2$

$$\frac{1}{1+e^{\beta'(\xi-\mu')}} \approx \frac{1}{1+e^{\beta'(1/2-\mu')}} - \frac{e^{\beta'(1/2-\mu')}}{(1+e^{\beta'(1/2-\mu')})^2}\beta'(\xi-1/2) + ((\beta'(\xi-1/2))^2). \qquad [33]$$

Replacing this expansion in Eq. (28) we get

$$\frac{1}{1+e^{\beta'(1/2-\mu')}} = \rho \implies \frac{\beta'}{2} - \beta'\mu' = \log\left(\frac{1-\rho}{\rho}\right). \qquad [34]$$

Replacing the expansion in Eq. (29) we get

$$\frac{\rho}{2} - \rho(1-\rho)\beta'\frac{1}{12} = \frac{\rho}{2} - \rho(1-\rho)(\langle AUC\rangle - \frac{1}{2}) \implies \beta' = 12(\langle AUC\rangle - \frac{1}{2}). \quad [35]$$

Combining Eqs. (34) and (35) we get the desired results:

$$\beta N = 12\left(\langle AUC\rangle - \frac{1}{2}\right) \quad [36]$$

$$\mu/N = \frac{1}{2} - \frac{1}{12\left(\langle AUC\rangle - \frac{1}{2}\right)}\log\left(\frac{1-\rho}{\rho}\right) \quad [37]$$

It is not difficult to verify that Eq. (37) results from Eq. (30) and Eq. (36) in the limit of $\beta \ll 1$. Note that when $\langle AUC\rangle \to 1/2$ (limit of random classifiers) the parameter $\beta \to 0$ (limit of infinite temperature). In this same limit, μ is undefined. However, the parameter $\beta\mu = 6\left(\langle AUC\rangle - 1/2\right) - \log((1-\rho)/\rho)$ is well defined and tends to $\log((1-\rho)/\rho)$ as $\langle AUC\rangle \to 1/2$.

B. Case $\beta \gg 1$. As β increases (or equivalently, as the temperature decreases), the $\langle AUC\rangle$ approaches 1. In this limit, the rank-conditioned class probability is a step function, taking the value of 1 when $r \leq \mu$ and 0 when $\mu < r \leq 1$. It is clear from Eq. (30) that in the limit of $\beta' \gg 1$ it is $\mu' = \rho$. Therefore, in this limit $\beta \gg 1$, $\mu = N_1$ and the rank-conditioned class probability close to the perfect classifier is

$$P(1|r) = \frac{1}{1 + e^{\beta(r-N_1)}}. \quad [38]$$

The slope of this distribution at $r = N_1$ is

$$\left.\frac{d}{dr}P(1|r)\right|_{r=N_1} = -\beta/4. \quad [39]$$

Given that in this limit the $P(1|r)$ is a steep logistic function, we will then approximate it with a linear function, where the slope of the line interpolating between 1 and 0 is determined by the slope of the actual distribution at $r = N_1$

$$P(1|r) \approx \begin{cases} 1 & \text{if } r \leq r_- \\ -\frac{\beta}{4}(r - N_1) + \frac{1}{2} & \text{if } r_- < r \leq r_+ \\ 0 & \text{if } r > r_+ \end{cases} \quad [40]$$

where $r_- = N_1 - 2/\beta$ and $r_+ = N_1 + 2/\beta$. Now we apply the constraint of Eq. (49)

$$N_1\frac{N+1}{2} + N_1(N-N_1)(\frac{1}{2} - \langle AUC\rangle) = \sum_{r=1}^{N} rP(1|r) \quad [41]$$

$$\approx \int_0^N rP(1|r)dr \quad [42]$$

$$\approx \int_0^{r_-} rdr + \int_{r_-}^{r_+} r\left[-\frac{\beta}{4}(r-N_1) + \frac{1}{2}\right]dr \quad [43]$$

$$= \frac{N_1^2}{2} + \frac{2}{3}\frac{1}{\beta^2}, \quad [44]$$

where we approximated the sum with an integral and used Eq.40. Solving for β from the last equation we find that close to the perfect classifier

$$\beta N = \sqrt{\frac{2}{3}}\frac{1}{\sqrt{\rho(1-\rho)(1-\langle AUC\rangle)}}$$

$$\mu/N = \rho.$$

C. Case $\rho = 1/2$. In this case, $\mu = N/2$ for any β. To show this, simply set $\rho = 1/2$ in Eq. (30).

D. Symmetries in the relations of β and μ as a function of $\langle AUC\rangle$ and ρ. Beyond the special cases discussed above, it is possible to find some symmetries that must hold for all $0 \leq \langle AUC\rangle \leq 1$ and $0 \leq \rho \leq 1$. Let us assume that a classifier g applied to an (N_1, N)-ensemble of test sets yields an average AUC $\langle AUC\rangle$ with parameters for $P(1|r)$ equal to $(\beta N, \mu/N) = \mathbf{f}(\rho, \langle AUC\rangle)$. It is not difficult to show that the following symmetries hold for function $\mathbf{f}$: $(-\beta N, 1-\mu/N) = \mathbf{f}(\rho, 1-\langle AUC\rangle)$ (which results if a classifier g' placed class 1 items with the same distribution that classifier g placed class 0 items), $(\beta N, 1-\mu/N) = \mathbf{f}(1-\rho, \langle AUC\rangle)$ (which results if class 0 items were now called class 1, and classifier g' ranked them with the opposite order than g does, e.g., $g' = -g$), and $(-\beta N, \mu/N) = \mathbf{f}(1-\rho, 1-\langle AUC\rangle)$ (the concatenation of the previous two operations). Some of the previous relations and symmetries can be easily seen in Figure (2) in the Main Text.

6. The relation between $\langle AUC \rangle$ and the average Wilcoxon-Mann-Whitney U statistics follows from the constraints imposed on the FD distribution

In the main text we recovered a result previously advanced in (3) stating that the AUC computed from the scores s_j outputted by a classifier from a finite test set using a rectangular integration rule can be written as

$$AUC = \frac{1}{N_1 N_0} \sum_{j|y_j=1} \sum_{i|y_i=0} \mathcal{H}(s_j - s_i), \tag{45}$$

where $\mathcal{H}$ is the Heaviside step function which is 1 if the argument is positive, 0 if negative and 1/2 if 0, and N_1 and N_0 are the number of positive and negative items in the test set. The double sum in Eq. (45) is known as the Mann-Whitney U statistics. As already discussed in the main text, if we take the average in the (N, N_1)-ensemble of test sets in both sides of Eq. (45), we get

$$\langle AUC \rangle = \mathrm{Prob}(s_j > s_i | y_j = 1; y_i = 0). \tag{46}$$

Next we show that Eq. (46) can be directly derived from the expressions of the $\langle \mathrm{TPR}(k) \rangle$ and $\langle \mathrm{FPR}(k) \rangle$ in terms of the rank-conditioned class probability as expressed in Eq. (2) and Eq. (3). Writing the $\langle AUC \rangle$ as the area of the average ROC in TPR vs FPR space using a rectangular integration rule, and then using Eqs. (2) and (3) we find that

$$\begin{aligned}
\langle AUC \rangle &= \sum_{i=1}^{N} \langle \mathrm{TPR}(i) \rangle \Delta \langle \mathrm{FPR}(i) \rangle \\
&= \frac{1}{N_1} \sum_{i=1}^{N} \sum_{r=1}^{i} P(1|r)(\langle \mathrm{FPR}(i+1) \rangle - \langle \mathrm{FPR}(i) \rangle) \\
&= \frac{1}{N_1 N_0} \sum_{i=1}^{N} \sum_{r=1}^{i} P(1|r) P(0|i+1) \\
&= \frac{1}{N_1 N_0} \sum_{r=1}^{N} \sum_{i=r+1}^{N} P(1|r) P(0|i).
\end{aligned} \tag{47}$$

Eq. (47) is equal to the probability that the rank of a negative item is larger than the rank of a positive item. This is so because if a positive item is at rank r, the probability that a negative item has a rank higher than r is $\sum_{i=r+1}^{N} P(i|0)$. As the positive item can be at any rank r, to compute $\mathrm{Prob}(r_j < r_i | y_j = 1; y_i = 0)$ we need to add the previous sum over all the possible ranks r where the positive item could be, weighted by the probability $P(r|1)$ that there is a positive item at rank r. Using that $P(r|1) = P(1|r)/N_1$ and $P(r|0) = P(0|r)/N_0$ we obtain Eq. (47).

As argued throughout this paper, the maximum entropy distribution for the rank-conditioned class probability $P(1|r)$ is given by the FD statistics: $P(y=1|r) = \frac{1}{1+e^{\beta(r-\mu)}}$ where the parameters β and μ are determined by the constraints given in Eqs. (48) and (49). Therefore it may seem that Eq. (47), which also links $P(1|r)$ and $\langle AUC \rangle$ will over-determine β and μ. We next show that this is not the case. Indeed Eq. (47) is valid for any rank-conditioned class probability function $P(y|r)$ that verifies the following constraints:

$$N_1 = \sum_{r=1}^{N} P(1|r), \tag{48}$$

$$N_1 \frac{N+1}{2} - N_1(N - N_1)(\langle AUC \rangle - \frac{1}{2}) = \sum_{r=1}^{N} r \ P(1|r). \tag{49}$$

For simplicity we will assume that $N \gg 1$, and rewrite the previous sums as integrals over the rescaled variable $x = r/N$

$$\rho = \int_0^1 dx \ P(1|x), \tag{50}$$

$$\frac{\rho}{2} - \rho(1-\rho)(\langle AUC \rangle - \frac{1}{2}) = \int_0^1 dx \ x \ P(1|x). \tag{51}$$

The replacement of sums by integrals imply that the results should be correct to within an error of the order of $1/N$. Let's now compute $\mathrm{Prob}(s_j > s_i | y_j = 1; y_i = 0)$ in terms of the probabilities of positive class given the rank

$$\mathrm{Prob}(s_j > s_i | y_j = 1; y_i = 0) = \frac{1}{\rho(1-\rho)} \int_0^1 dx \ P(1|x) \int_x^1 dy \, (1 - P(1|y)) \tag{52}$$

$$= \frac{1}{\rho(1-\rho)} \left(\int_0^1 dx \ (1-x) \ P(1|x) - \int_0^1 dx \ P(1|x) \int_x^1 dy P(1|y) \right). \tag{53}$$

The first integral in Eq. (53) can be easily found from Eq. (50) and Eq. (51):

$$\frac{1}{\rho(1-\rho)}\left(\int_0^1 dx\ P(1|x) - \int_0^1 dx\ x\ P(1|x)\right) = \langle AUC\rangle + \frac{\rho}{2(1-\rho)}. \quad [54]$$

The second double integral in Eq. (53) can be solved by multiplying Eq. (50) by itself and doing some algebra:

$$\rho^2 = \int_0^1 dx\ P(1|x) \int_0^1 dy\ P(1|y) \quad [55]$$

$$= \int_0^1 dx\ P(1|x) \int_0^x dy\ P(1|y) + \int_0^1 dx\ P(1|x) \int_x^1 dy\ P(1|y) \quad [56]$$

$$= 2\int_0^1 dx\ P(1|x) \int_x^1 dy\ P(1|y), \quad [57]$$

where we used that, because of symmetry, it is

$$\int_0^1 dx\ P(1|x) \int_0^x dy\ P(1|y) = \int_0^1 dx\ P(1|x) \int_x^1 dy\ P(1|y),$$

and therefore

$$\int_0^1 dx\ P(1|x) \int_x^1 dy\ P(1|y) = \frac{\rho}{2}. \quad [58]$$

Inserting Eq. (54) and Eq. (58) into Eq. (53) we get

$$\text{Prob}(s_j > s_i | y_j = 1; y_i = 0) = \langle AUC\rangle. \quad [59]$$

as desired.

7. Derivation of the expression for the variance of the AUC given in the main text

As discussed in (4), the variance of the AUC of a classifier can be computed using Eq. (45). To do that we recall that for any random variable X, $\sigma_X^2 = \langle X^2\rangle - \langle X\rangle^2$, which we will apply to the AUC. We first compute AUC^2 by squaring Eq. (45), from where we get:

$$AUC^2 = \frac{1}{N_1^2 N_0^2}\sum_{i=1}^{N_1}\sum_{k=1}^{N_0}\sum_{j=1}^{N_1}\sum_{m=1}^{N_0}\mathcal{H}(s_{P,i}-s_{N,k})^2\mathcal{H}(s_{P,j}-s_{N,m})$$

$$= \frac{1}{N_1^2 N_0^2}\Bigg[\sum_{i=1}^{N_1}\sum_{k=1}^{N_0}\mathcal{H}(s_{P,i}-s_{N,k}) + \sum_{i=1}^{N_1}\sum_{k=1}^{N_0}\sum_{\substack{m=1\\ m\neq k}}^{N_0}\mathcal{H}(s_{P,i}-s_{N,k})\mathcal{H}(s_{P,i}-s_{N,m})$$

$$+ \sum_{i=1}^{N_1}\sum_{\substack{j=1\\ j\neq i}}^{N_1}\sum_{k=1}^{N_0}\mathcal{H}(s_{P,i}-s_{N,k})\mathcal{H}(s_{P,j}-s_{N,k}) + \sum_{i=1}^{N_1}\sum_{k=1}^{N_0}\sum_{\substack{j=1\\ j\neq i}}^{N_1}\sum_{\substack{m=1\\ m\neq k}}^{N_0}\mathcal{H}(s_{P,i}-s_{N,k})\mathcal{H}(s_{P,j}-s_{N,m})\Bigg] \quad [60]$$

where $s_{P,i}$ and $s_{N,k}$ are the scores assigned by the classifier to the i-th positive item and the k-th negative example respectively and $\mathcal{H}(\int)$ is the Heaviside function which takes the value of 1 for $s > 0$ and 0 for $s < 0$. For the sake of simplicity we will assume that there are no ties in the scores, which is a reasonable assumption if the scores lie on a continuous scale. Therefore $\mathcal{H}(s_{P,i}-s_{N,k})^2 = \mathcal{H}(s_{P,i}-s_{N,k})$. Taking the expected value in the above equation over the $(N, N_1)-$ensembles of test sets we get

$$\langle AUC^2\rangle = \frac{1}{N_1^2 N_0^2}\Big[N_1 N_0\langle\mathcal{H}(s_{P,i}-s_{N,k})\rangle + N_0(N_0-1)N_1\langle\mathcal{H}(s_{P,i}-s_{N,k})\mathcal{H}(s_{P,i}-s_{N,m})\rangle$$

$$+ N_0 N_1(N_1-1)\langle\mathcal{H}(s_{P,i}-s_{N,k})\mathcal{H}(s_{P,j}-s_{N,k})\rangle \quad [61]$$

$$+ N_0(N_0-1)N_1(N_1-1)\langle\mathcal{H}(s_{P,i}-s_{N,k})\rangle\langle\mathcal{H}(s_{P,j}-s_{N,m})\rangle\Big]. \quad [62]$$

Note that $\langle\mathcal{H}(s_{P,i}-s_{N,k})\rangle$ is the probability that a positive item has a score larger than a negative item, which was earlier shown to be equal to $\langle AUC\rangle$ (see Eq. (46)). We will call

$$P_{100} = \langle\mathcal{H}(s_{P,i}-s_{N,k})\mathcal{H}(s_{P,i}-s_{N,m})\rangle$$
$$P_{110} = \langle\mathcal{H}(s_{P,i}-s_{N,k})\mathcal{H}(s_{P,j}-s_{N,k})\rangle.$$

P_{100} is the expected value of $\mathcal{H}(s_{P,i} - s_{N,k})\mathcal{H}(s_{P,i} - s_{N,m})$, which is 1 when the two randomly sampled negative items $s_{N,k}$ and $s_{N,m}$ have a score that is lower than a randomly sampled positive item $s_{P,i}$ and 0 otherwise. Therefore P_{100} is the probability that the classifier assigns two randomly sampled negative items scores that are smaller than the score assigned to a randomly sampled positive item. Similarly P_{110} is the expected value of $\mathcal{H}(s_{P,i} - s_{N,k})\mathcal{H}(s_{P,j} - s_{N,k})$, which is 1 when the two randomly sampled positive items $s_{P,i}$ and $s_{P,j}$ have scores that are larger than a randomly sampled negative item $s_{N,k}$ and 0 otherwise. Therefore P_{110} is the probability that the classifier assigns two randomly sampled positive items scores that are larger than the score assigned to a randomly sampled negative item. Assembling these results, σ^2_{AUC} can be written as

$$\begin{aligned}\sigma^2_{AUC} &= \langle AUC^2 \rangle - \langle AUC \rangle^2 \\ &= \frac{1}{N_1 N_0}\Big[\langle AUC \rangle + (N_0 - 1)P_{100} + (N_1 - 1)P_{110} + (N_0 - 1)(N_1 - 1)\langle AUC \rangle^2 - N_0 N_1 \langle AUC \rangle^2\Big] \\ &= \frac{1}{N_1 N_0}\Big[\langle AUC \rangle(1 - \langle AUC \rangle) + (N_0 - 1)(P_{100} - \langle AUC \rangle) + (N_1 - 1)(P_{110} - \langle AUC \rangle)\Big].\end{aligned} \tag{63}$$

Eq. (63) is the expression for the variance of the AUC that we used in the main text.

8. Ensemble Classifier performance with conditionally correlated rank predictions

The FiDEL ensemble was derived under the assumption that base classifier predictions are class conditionally independent. In this section we use class conditionally dependent simulation data to empirically assess the extent to which class conditional dependence influences the performance of the FiDEL ensemble. The base classifiers will be generated to span a range of AUC's. We compare the performance of the FiDEL ensemble to the best individual synthetic base classifier and a naive ensemble that we will call the Wisdom-of-Crowds (WOC) (5) constructed by the unweighted mean of base classifier rank predictions. The simulations and classifiers were implemented using the Python programming language and common packages within the SciPy ecosystem (6–8).

Simulation data were prepared by rank transforming sample scores drawn from two M dimensional multivariate Gaussian distributions, with each of these two multivariate Gaussians corresponding to one of the two classes. The AUC of each of the M base classifiers and the class conditional dependence of their scores were set by choosing the difference of the distribution means and their covariance matrices, respectively.

We first choose the target performance AUC_i of base classifier $i \in \{1, 2, \ldots, M\}$ as $AUC_i = 0.55 + 0.2(i-1)/(M-1)$, spanning a range between 0.55 and 0.75. Without loss of generality we will assume that the mean of the scores of the positive class of all the classifiers is $\mu_{i|Y=1} = 0$. Therefore, the ith conditional mean of the negative class $\mu_{i|Y=0}$ can be computed from its AUC_i and the diagonal elements of the class-conditioned covariance matrices Σ_Y by

$$\mu_{i|Y=0} = \sqrt{\Sigma_{ii|Y=0} + \Sigma_{ii|Y=1}}\,\Phi^{-1}(AUC_i) \tag{64}$$

as defined in (9) with Φ^{-1} being the inverse of the standard normal cumulative. As in the main paper we use the convention that ranks assigned by better than random classifiers (i.e., $AUC > 0.5$) are such that positive class items get ranks that will be lower in average than the ranks of the negative class items. Ranks were produced from the scores of the base classifiers using the rank transform algorithm (within the stats module of the scipy package, (7)) assigning low and high sample scores, low and high sample ranks, respectively.

For the purpose of determining the effect of the conditional dependence on the performance of FiDEL, we will limit the class conditioned covariance matrices of the base classifier scores to the form of:

$$\Sigma_{ij|Y=1} = \Sigma_{ij|Y=0} = \begin{cases} 1 & \text{for } i = j, \\ r & \text{for } i \neq j \text{ and } 0 \leq r < 1. \end{cases} \tag{65}$$

As Eq. (65) specifies that the diagonal entries are 1 and that $0 \leq r < 1$, we may then interpret the class conditioned covariance matrix $\mathbf{\Sigma}_Y$ as a class conditioned correlation matrix and r the class conditioned correlation coefficient of the classifier scores. With Eq. (64) and Eq. (65) we generate sample scores by specifying the number of samples N, the prevalence of the positive class, AUC_i for $i \in \{1, 2, \ldots, M\}$, and the class conditioned score correlation coefficient r. Note that even though we have a target AUC_i for classifier i, the empirical $\hat{AUC}_i$ will be slightly different from its intended target due to sampling variations. Similarly, while we set a target class conditioned score covariance matrix with off-diagonal terms equal to r, we will report the average class conditioned rank correlation coefficient $\hat{r}$ over all $M(M-1)/2$ pairs of base classifiers and over the two classes. It turns out that the target class conditioned score correlation r and the average of the empirical class conditioned rank correlations $\hat{r}$ are numerically similar.

Using the outlined data simulation strategy we generated synthetic data for distinct values of M and r. With these synthetic data we compute the β_i and μ_i of each classifier, based on the empirical AUC_i and the prevalence of the positive class to generate the FiDEL ensemble model. We then assess the empirical training performance (AUC) of FiDEL, WOC, and the best individual base classifier for increasing values of r. In this way we aim to establish the dependence of FiDEL performance on the class conditioned rank correlation $\hat{r}$. The results are presented in Figures S1 and S2.

We first establish the relative performance of FiDEL, WOC, and the best individual base classifier when $r = 0$ (and therefore $\hat{r} = 0$), a case in which our assumption of class conditional independence holds. Figure S1A shows that an increase in the

number of base classifiers corresponds to a monotonic increase in the empirical performance of FiDEL and WOC ensemble classifiers, with FiDEL showing a performance that is slightly better than the WOC ensemble. The trend presented in these empirical results suggest that for relatively large numbers of base classifiers, the performance of FiDEL and WOC are far superior than any one of their base classifier constituents, converging asymptotically to an AUC of 1.

Next, we examine the influence that conditionally dependent base classifier predictions have on the performance of FiDEL and WOC ensemble classifiers. Figure S1B ($\hat{r} = 0.19$) suggests that the AUC of FiDEL and WOC reach distinct performance maxima, each of which are less than the maximum AUC reported for conditionally independent base classifier predictions in Figure S1A. In addition, these results show that the performance of the best individual method is greater than or equal to that of the WOC ensemble classifier for $M \leq 7$. Distinctly, the performance of the FiDEL ensemble classifier is greater than that of the WOC and it is clearly better than the best individual classifier for all $M > 2$. With an increase in the class conditional dependence to $\hat{r}$=0.38, the performances of the ensemble classifiers continue to decline. Figure S1C suggests that the performance of FiDEL is close to that of the best individual base classifier, but better than that of the WOC. The WOC performance, on the other hand, is worse than the best individual base classifier for all M. A further increase of conditional correlation to $\hat{r}$=0.58 in Figure S1D results in an empirical AUC value of each ensemble that is less than the best individual base classifier for all M tested. These results are summarized for $M = 10$ in Figure S2, and together suggest that the performance of FiDEL is robust to mild violations in the assumption of class conditional independence and its performance is greater than the best individual classifiers up to a class conditioned correlation of $\hat{r} \lesssim$0.4, and is better than the WOC ensemble for all values of $\hat{r}$ tested.

9. Datasets and classifiers

To exemplify the use of FiDEL we leveraged two datasets previously used in two Kaggle competitions: The West Nile Virus (WNV) Prediction challenge (10) and the Springleaf Marketing Response (SMR) challenge (11) (Table S1). For the WNV dataset we used all the 10,506 items available, but deleted several features that contained outliers. This reduced the feature set from 85 to 75. For the SMR dataset we sub-selected 22,000 items chosen randomly out of the original training data set of 145,231 items. This was done to reduce the computational burden and memory usage required to train some of the algorithms. We also removed features with missing values (N/A or specific codes to mark the unavailable values such as 9X, 99X, 999X, 99999999X). This led to a reduced feature set of 259 features compared to the original 1,934 features in the SMR data.

The processing of the datasets as well as the results of Figure 4 can be reproduced using the code in https://github.com/sungcheolkim78/FiDEL/tree/master/kaggle and https://github.com/sungcheolkim78/FiDEL respectively. This code has the random seed fixed as 200 (set.seed(200)) to ensure reproducibility. However, it should be mentioned that if the random seed were changed, different training and test set partitions would be created, and different algorithms would be assigned to the new partitions, resulting in slightly different performances for FiDEL than the ones reported in Figure 4 of the main paper.

The classification methods used to create FiDEL ensembles are shown in Table S2. There are a total of 23 methods which were selected to cover a broad range of algorithms. Not all methods were compatible with the features of each data set. Table S2 enumerates the 21 and 20 methods applied to the WNV and SMR data sets, respectively.

Table S1. Datasets Used to Evaluate FiDEL

Name	Items (Used)	Feature (Used)	ρ	Source
West Nile Virus (WNV)	10,506 (10,506)	85 (75)	0.08	Kaggle
Springleaf Marketing Response (SMR)	145,231 (22,000)	1934 (258)	0.24	Kaggle

Table S2. Machine learning methods used for FiDEL Combinations

Name	Main Method	RLibrary	WNV	SMR
avNNet	Model Averaged Neural Network	nnet	Yes	Yes
bayesglm	Bayesian Generalized Linear Model	arm	Yes	Yes
ctree	Conditional Inference Tree	party	Yes	Yes
C5.0	Decision Trees and Rule-Based Models	C50	No	Yes
dwdRadial	Distance Weighted Discrimination	kerndwd	Yes	Yes
earth	Multivariate Adaptive Regression Spline	earth	Yes	Yes
gbm	Stochastic Gradient Boosting	gbm	Yes	Yes
glm	Generalized Linear Model	stats	Yes	Yes
glmnet	Lasso and Elastic-Net Regularized Generalized Linear Models	glmnet	Yes	Yes
knn	k-Nearest Neighbors	caret	Yes	No
rotationForest	Rotation Forest	rotationForest	Yes	No
nnet	Neural Network	nnet	Yes	Yes
mlp	Multi-Layer Perceptron	RSNNS	Yes	Yes
pls	Partial least squares regression	pls	Yes	Yes
rda	Regularized Discriminant Analysis	klaR	Yes	Yes
rmda	Robust Mixture Discriminant Analysis	robustDA	No	Yes
rf	Random Forest	randomForest	Yes	Yes
rpart	Recursive Partitioning and Regression Trees	rpart	Yes	No
simpls	Partial Least Squares	pls	Yes	Yes
svmLinear	Support Vector Machine with Linear Kernel	kernlab	Yes	Yes
svmRadial	Support Vector Machine with Radial Kernel	kernlab	Yes	Yes
xgbLinear	eXtreme Gradient Boosting	xgboost	Yes	Yes
xgbTree	eXtreme Gradient Boosting	xgboost	Yes	Yes

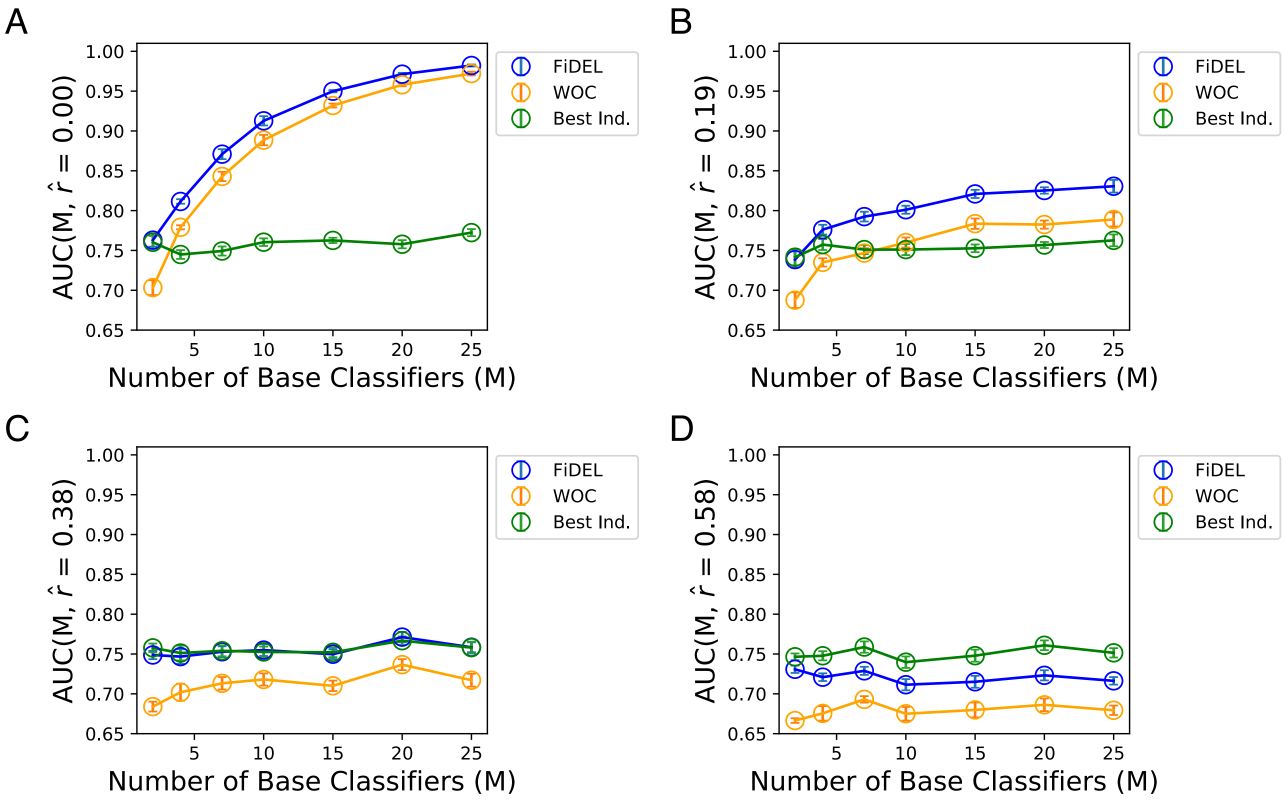

Fig. S1. The dependence of the average AUC $\pm$ standard error of the mean for N_{reps} replicas of the FiDEL, WOC, and the best individual synthetic classifier as a function of the number of base classifiers M=2, 4, 7, 10, 15, 20, and 25, given different class conditioned rank correlation coefficients $\hat{r}$. For each pair of parameters (M, r), where r is the target class conditioned score correlation coefficient between pairs of classifiers, we conducted N_{reps}=10 replicate simulation experiments. For each simulation experiment we set the total number of samples N=500, the prevalence of the positive class labels to 0.4, and base classifier target AUC values were uniformly selected on the interval [0.55, 0.75]. The empirical average class conditioned rank correlation coefficient between pairs of classifiers $\hat{r}$ were computed to be (A) 0, (B) 0.19, (C) 0.38, and (D) 0.58.

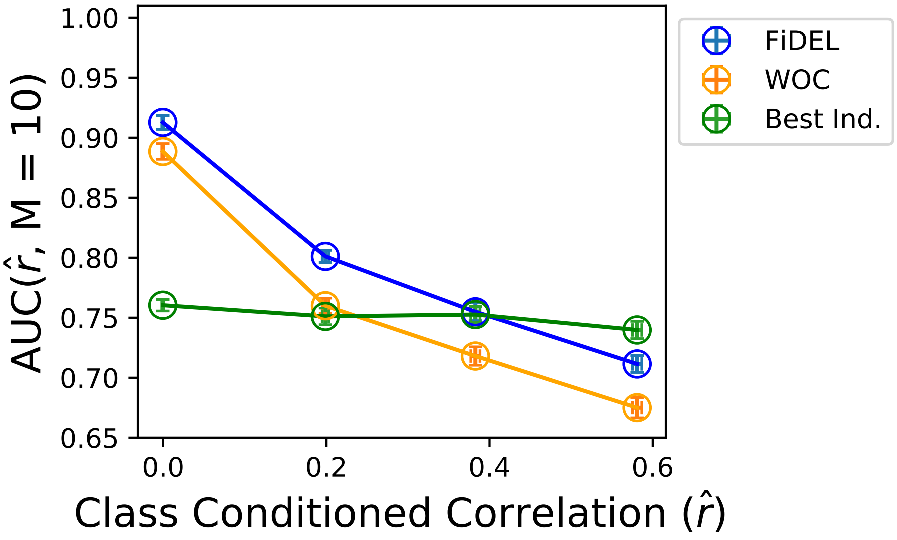

Fig. S2. The dependence of the average AUC $\pm$ standard error of the mean for N_{reps} replicas of the FiDEL, WOC, and the best individual classifier as a function of the average $\hat{r} \pm$ standard error of the mean for N_{reps} replicas. For each target value of the class conditioned score correlation coefficient between pairs of classifiers r=0, 0.2, 0.4 and 0.6 we conducted N_{reps}=10 replicate simulation experiments. For each replicate simulation we set the total number of samples N=500, the prevalence of the positive class to 0.4, M=10 base classifiers with AUC values uniformly selected on the interval [0.55, 0.75], and computed the average of the empirical class conditioned rank correlation coefficient $\hat{r}$. More precisely $\hat{r}$ is the average over all $M(M-1)/2$ pairs of classifiers and over the two classes, of the empirical class conditioned rank correlation coefficient.

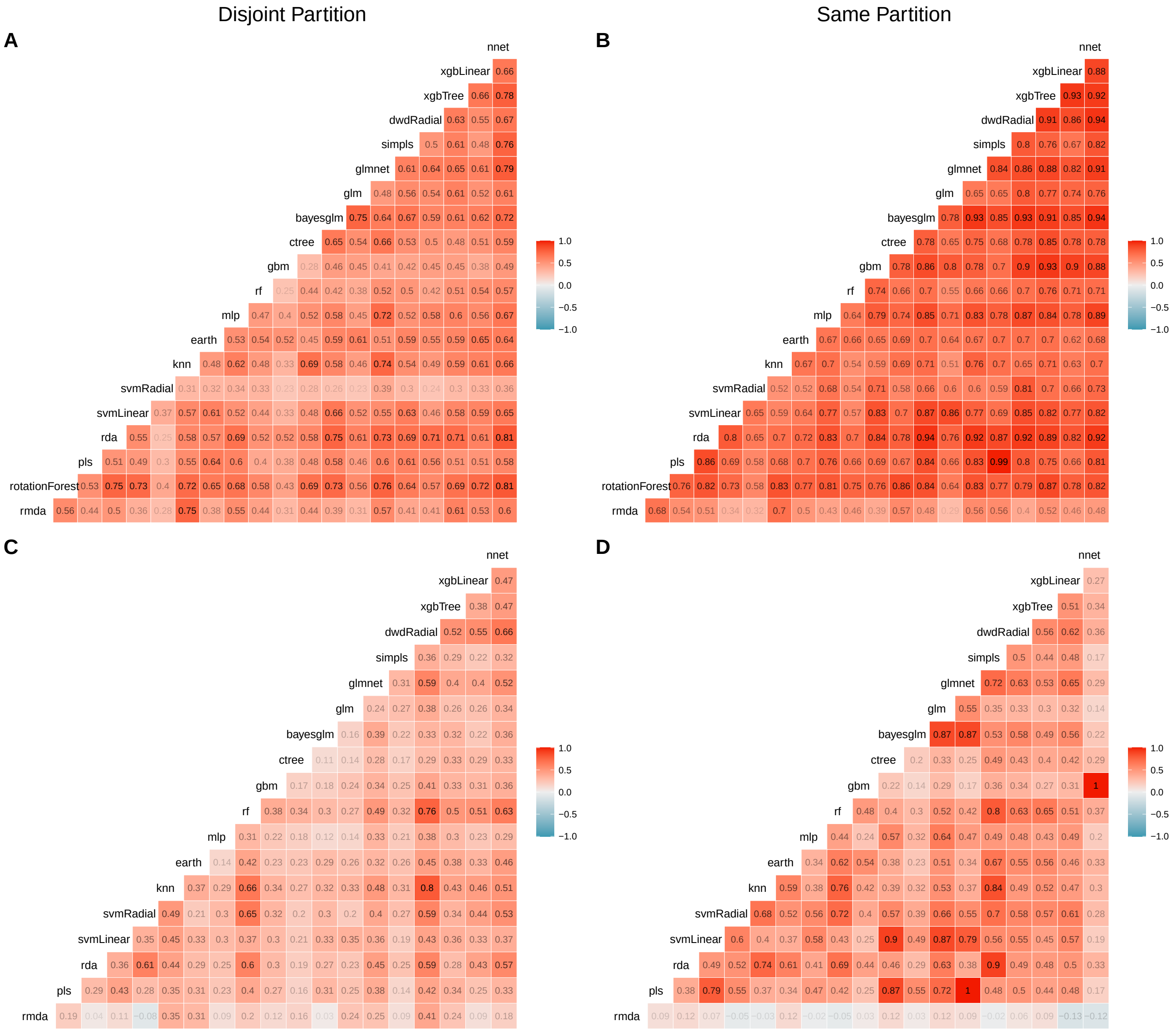

Fig. S3. The average over the positive and negative class conditioned correlation of all pairs of base classifier rank predictions in the test set, using two different training protocols: the "Disjoint Partition" strategy (A,C) in which each classifier was trained in a different partition of the training set and the "Same Partition" strategy (B,D) in which one partition of the training set was chosen to train all classifiers. (A) West Nile Virus dataset where each of the 21 base classifiers were trained using the Disjoint Partition strategy. Mean correlation is $\hat{r}$=0.54 with a standard deviation σ of 0.13. (B) West Nile Virus dataset where each of the 21 base classifiers were trained using the Same Partition strategy. $\hat{r}$=0.66 and σ=0.13. (C) Springleaf Marketing Response dataset where each of the 20 base classifiers were trained using the Disjoint Partition strategy. $\hat{r}$=0.32 and σ=0.14. (D) Springleaf Marketing Response dataset where each of the 20 base classifiers were trained using the the Same Partition strategy. $\hat{r}$=0.44 and σ=0.21. The C5.0 classifier was uninformative, with an AUC of 0.5 in both datasets, and hence, it is not included in the above correlation plots. The Disjoint Partition strategy reduces the correlation between predictions in the test set compared to the Same Partition strategy.

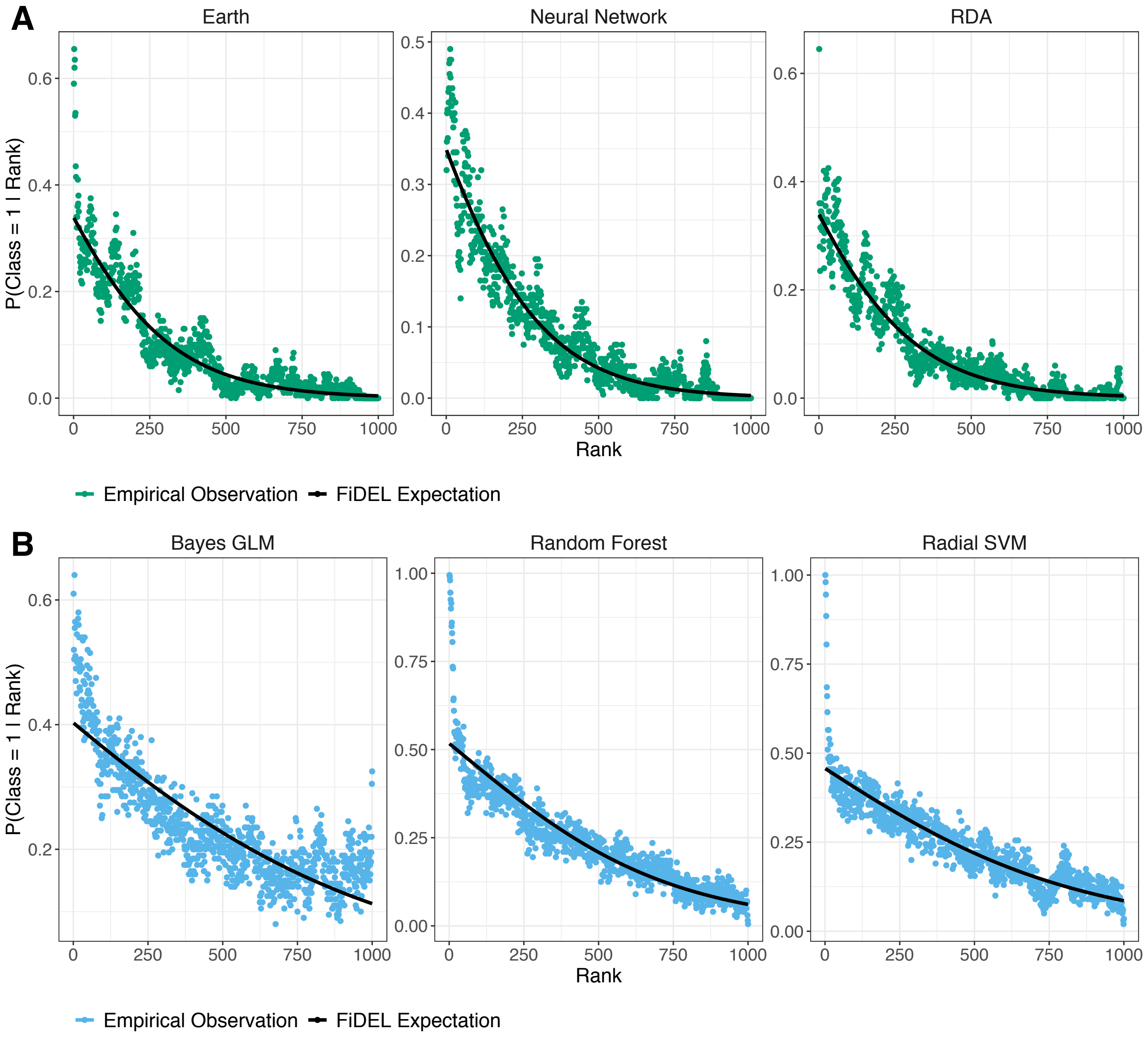

Fig. S4. Empirical estimation of the rank-conditioned positive class probability $P(class = 1|rank)$ (blue and green circles) and the corresponding FD distribution fit for the WNV and SMR datasets and 6 different base classifiers. The classifiers used in this figure were trained using the same partition as in the Disjoint Partition training strategy used for Fig. S3. The estimation of $P(class = 1|rank)$ was done by sampling 200 randomly chosen subsets selected amongst the training set partition not used to train the classifier. Each subset had N=1000 items. The items in each subset were ranked and the class label (1 or 0) of the item placed at rank r was stored. $P(class = 1|rank = r)$ was estimated as the fraction of class 1 items that were placed at rank r. The solid lines in each of the sub-figures are the FD distribution fit using the prevalence ρ and the AUC of the classifier computed using the complement of the training set not used to train the classifier. (A) Results for the WNV dataset (ρ= 0.08) and three classifiers: Earth, Neural Network and RDA. Earth is a multivariate adaptive regression spline method with an AUC = 0.726. The resulting FD distribution parameters are β = 4.81 and μ = -0.14. The Neural Network classifier had an AUC = 0.75, and FD distribution parameters β = 5 and μ = -0.12. RDA, a regularized discriminant analysis classifier, had an AUC= 0.727, and FD distribution parameters β = 4.83 and μ = -0.14. (B) Results for the SMR dataset and three classifiers: Bayes GLM, Random Forest and Radial SVM. The Bayes GLM classifier, had am AUC=0.671, and FD distribution parameters β = 1.67 and μ = 0.24. The Random Forest classifier had an AUC=0.742 and FD distribution parameters β = 2.81 and μ = 0.025. The Radial SVM (Support Vector Machine) classifier had an AUC = 0.685 and FD distribution parameters β = 2.20, μ = -0.078. The fitted FD distributions closely approximate the trends of the empirical $P(class = 1|rank)$ for the classifiers used in this figure, and are representative of the other classifiers not shown in the figure.

References

1. ME Ahsen, RM Vogel, GA Stolovitzky, Unsupervised evaluation and weighted aggregation of ranked classification predictions. *J. Mach. Learn. Res.* **20**, 1–40 (2019).
2. ET Jaynes, Information theory and statistical mechanics. *Phys. Rev.* **106**, 620–630 (1957).
3. SJ Mason, NE Graham, Areas beneath the relative operating characteristics (roc) and relative operating levels (rol) curves: Statistical significance and interpretation. *Q. J. Royal Meteorol. Soc. A journal atmospheric sciences, applied meteorology physical oceanography* **128**, 2145–2166 (2002).
4. JA Hanley, BJ McNeil, The meaning and use of the area under a receiver operating characteristic (roc) curve. *Radiology* **143**, 29–36 (1982).
5. D Marbach, et al., Wisdom of crowds for robust gene network inference. *Nat. methods* **9**, 796–804 (2012).
6. CR Harris, et al., Array programming with numpy. *Nature* **585**, 357–362 (2020).
7. P Virtanen, et al., Scipy 1.0: fundamental algorithms for scientific computing in python. *Nat. methods* **17**, 261–272 (2020).
8. JD Hunter, Matplotlib: A 2d graphics environment. *Comput. Sci. & Eng.* **9**, 90–95 (2007).
9. C Marzban, The roc curve and the area under it as performance measures. *Weather. Forecast.* **19**, 1106–1114 (2004).
10. The west nile virus prediction challenge (2015) Available at: https://www.kaggle.com/c/predict-west-nile-virus [Accessed December 26, 2020].
11. The springleaf marketing response challenge (2015) Available at: https://www.kaggle.com/c/springleaf-marketing-response [Accessed December 26, 2020].
